# Analysis of early childhood intestinal microbial dynamics in a continuous-flow bioreactor

**DOI:** 10.1186/s40168-024-01976-w

**Published:** 2024-12-05

**Authors:** Alessandra Granato, Simone Renwick, Christopher Yau, Tiffany Kong, Michelle C. Daigneault, Mikael Knip, Emma Allen-Vercoe, Jayne S. Danska

**Affiliations:** 1 Genetics and Genome Biology, The Hospital for Sick Children, Toronto, ON Canada; 2 Dept. of Molecular and Cellular Biology, University of Guelph, Guelph, ON Canada; 3 Infant Center of Research Excellence, The Larsson-Rosenquist Foundation Mother-Milk, University of California San Diego, La Jolla, San Diego, CA USA; 4 Dept. of Immunology, Faculty of Medicine, University of Toronto, Toronto, ON Canada; 5 Research Program for Clinical and Molecular Metabolism, Faculty of Medicine, University of Helsinki, Helsinki, Finland; 6 Tampere Center for Child Health Research, Tampere University Hospital, Tampere, Finland; 7 Dept. of Medicine Biophysics, Faculty of Medicine, University of Toronto, Toronto, ON Canada

**Keywords:** Early childhood microbiome, Chemostat continuous culture, Bacterial metabolites, Anexic isolation, Defined bacterial consortia

## Abstract

**Background:**

The human gut microbiota is inoculated at birth and undergoes a process of assembly and diversification during the first few years of life. Studies in mice and humans have revealed associations between the early-life gut microbiome and future susceptibility to immune and metabolic diseases. To resolve microbe and host contributing factors to early-life development and to disease states requires experimental platforms that support reproducible, longitudinal, and high-content analyses.

**Results:**

Here, we deployed a continuous single-stage chemostat culture model of the human distal gut to study gut microbiota from 18- to 24-month-old children integrating both culture-dependent and -independent methods. Chemostat cultures recapitulated multiple aspects of the fecal microbial ecosystem enabling investigation of relationships between bacterial strains and metabolic function, as well as a resource from which we isolated and curated a diverse library of early life bacterial strains.

**Conclusions:**

We report the reproducible, longitudinal dynamics of early-life bacterial communities cultured in an advanced model of the human gut providing an experimental approach and a characterized bacterial resource to support future investigations of the human gut microbiota in early childhood.

**Supplementary Information:**

The online version contains supplementary material available at 10.1186/s40168-024-01976-w.

## Background

The infant intestinal microbiota is inoculated at birth and undergoes a process of assembly throughout the first 6 months [1–5], followed by continuing diversification and stabilization of the microbial community over the first 2–3 years of life [6–11]. A growing body of research in mouse models and longitudinal human cohort studies have revealed associations between the assembly and function of the early life gut microbiota and susceptibility to the later development of immune-mediated and metabolic diseases [12–20]. Whole metagenome sequence analysis of human fecal samples has advanced our understanding of the inferred functional complexity of these microbial communities; however, inter-individual and interpopulation variability, methodological processes including fecal sampling, storage, and DNA extraction continue to limit the generalization of findings across studies. Innovative experimental platforms that support reproducible, longitudinal analysis and integration of analytical technologies are needed to advance a comprehensive understanding of the infant gut microbiota and to support clinical applications of microbiome research.

Characterization of the infant gut microbiota has advanced through culture-independent methods, including fecal 16S *rRNA* gene and whole shotgun metagenome sequencing and metabolomic analyses deployed in large, longitudinal cohort studies such as DIABIMMUNE [16, 21], TEDDY [7], and CHILD [22]. These methods have provided valuable insights into the taxonomic composition, diversity, and developmental trajectory of early life fecal community assembly. Reliance on human fecal sampling has attendant limitations in capturing the dynamics and functional activity of the gut microbial community. Identification of microbial contributions by fecal sample analysis is confounded by complex host-microbe interactions. To address these issues, in vitro models have been devised to emulate the gut microbiota by culturing microbes in a controlled environment [23, 24]. Multiple in vitro culture models have been developed for the study of the human gut microbiota [25–41]. Several have been applied to microbiota from young children to study impacts of pre-biotics [33, 42]. These models vary in complexity and capacity to emulation a complex intestinal ecosystem, including nutritional conditions, oxygen levels, pH, temperature, and transit time [24, 36]. The single-stage chemostat “Robogut” system employed here models the distal colon rather than the entire gastrointestinal tract [26]. In contrast to fed-batch systems, the chemostat is operated under continuous fermentation conditions to closely represent physiological conditions of the colonic ecosystem. In addition to culture models for complex microbiota, high-throughput axenic culture, where individual microorganisms are cultured in isolation, have been deployed to study functional properties of gut bacterial species [43–46], but do not capture community behavior. Collectively, the distinct features of these different approaches highlight the need for complementary and integrated approaches to address the assembly and functions of the gut microbiota in early life.

In this study, we employed a continuous single-stage chemostat culture model of the human distal gut [25, 47] to study the infant gut microbiota with integrated culture-dependent and -independent approaches. We cultivated and characterized the taxonomic composition and metabolic output of gut bacterial communities derived from healthy children in the first 18–24 months of life in a host-free chemostat system, together with axenic culture methods to generate a diverse archive of gut bacterial strains. The chemostat culture approach provided a system complementary to both culture-independent and traditional microbiological culture methods providing a representative and reproducible platform to advance a comprehensive understanding of the early childhood gut microbiome.

## Methods

### Study cohort and sample collection

Fecal samples were collected by participants’ parents enrolled in the DIABIMMUNE infant cohort [16] and stored in the household freezer (− 20 °C) until the next visit to the local study center; samples were then shipped on dry ice and stored at − 80 °C until use. Infant donors were selected based on the following inclusion criteria: no signs of illness, age between 18 and 24 months, solid food diet, and no antibiotics or any other drug intake. A portion of the selected fecal samples were shipped to the Allen-Vercoe lab under the University of Guelph REB no. 17–06-006 for chemostat culture.

### Chemostat culture medium

Chemostat media was developed by McDonald et al. [47] to propagate the human gut microbiota in bioreactor vessels. This formulation was designed to mimic the result of the predigestion of solid food by the upper gastrointestinal tract. Mucin was included as it is the main structural component of the mucus secreted by the goblet cells of the intestinal epithelium. Components of the chemostat medium are listed in Supplementary Table 1.

### Fecal-derived chemostat culture

Fecal slurries were prepared under anaerobic conditions (10% H_2_, 10% CO_2_, balanced with N_2_) in an anaerobe chamber (AS-580, Anaerobe Systems, Morgan Hill, CA, USA). Briefly, frozen fecal samples (0.6–1 g) were partially thawed and transferred into sterile bags containing 10 mL of sterile degassed chemostat medium. The mixtures were homogenized by hand to form slurries. Each fecal slurry was used to inoculate a 400-mL Multifors bioreactor system vessel (Infors AG, Bottmingen/Basel, Switzerland) already containing 390-mL sterile degassed chemostat media at 37 °C, pH 7, and stirring at 50 rpm [26]. Vessels were maintained under anaerobic, batch fermentation conditions for 48 h before switching to continuous fermentation conditions. Chemostat medium was fed at a flow rate of 400 mL/day to ensure a 24-h turnover of vessel contents. Vessels were maintained for 21–28 days. Daily samples (5 × 2-mL aliquots) were collected and stored at − 80 °C for subsequent experiments.

### Library of infant gut bacterial strains

Fecal-derived bacterial strains were isolated as pure cultures from aliquots of chemostat culture collected on days 1 and 28 from the NS1 fecal-derived community or NS0 fecal-derived community. Fresh cultures of 500 μL were serially diluted in sterile, degassed tryptic soy broth (Millipore Sigma, Burlington, MA, USA) supplemented with 5 μg/mL hemin and 1 μg/mL menadione in an anaerobe chamber under anaerobic conditions as above. Briefly, aliquots of chemostat culture were serially diluted, and 100 μL of dilutions (10^−4^, 10^−5^, 10^−6^, and 10^−7^) was spread-plated on the listed media types (Suppl. Table 2). Agar plates were incubated at 37 °C under aerobic and anaerobic conditions for 1 week. Over the following 5 days, individual colonies were selected based on varying morphologies and streak purified on fastidious anaerobe agar (Neogen, Lexington, KY, USA), supplemented with defibrinated 5% sheep’s blood (HemoStat Laboratories, Dixon, CA, USA; FAA) patch plates. Isolates were identified by first amplifying the V3–V6 region of their 16S rRNA gene using 1 µL of biomass and the primers V3kl (5′-TACGG[AG]AGGCAGCAG-3′) and V6r (5′-AC[AG]ACACGAGCTGACGAC-3′). Polymerase chain reaction (PCR) cycling conditions were 95 °C for 15 min, followed by 30 cycles of 94 °C for 2 min, 94 °C for 30 s, 60 °C for 30 s, and 72 °C for 30 s, with a final elongation at 72 °C for 5 min. PCR amplicon were fluorescently tagged using a BigDye Terminator v3.1 Cycle Sequencing Kit (Applied Biosystems, ThermoFisher Scientific, Waltham, MA, USA). T7 forward primer (5′-TAATACGACTCACTATAGGG-3′) and 25 cycles of 96 °C for 5 min, 96 °C for 30 s, 50 °C for 15 s, and 60 °C for 2 min were used for the fluorescent tagging by the BigDye Terminator. Resulting samples underwent Sanger sequencing performed by the Advanced Analysis Centre (AAC) Genomics Facility at the University of Guelph. Isolates were identified by cross-referencing against the NCBI 16S rRNA gene sequence reference database using the NCBI BLASTn tool (https:/www.ncbi.nlm.nih.gov/BLAST/). Pure cultures of unique strains (library of isolates) were archived in freezing medium (12% skim milk, 1% glycerol, and 1% DMSO) at − 80 °C.

### Defined chemostat communities

Defined communities were assembled from the library of gut bacterial isolates. Isolates were revived from frozen stocks by streaking partially thawed culture onto degassed fastidious anaerobic agar (Lansing, MI, USA) supplemented with defibrinated 5% sheep’s blood (FAA) plates under anaerobic conditions. After 3 days, isolates were streaked onto additional FAA plates and cultured for three additional days. Equal biomass by volume (one 10-μL microbiological loop) of each isolate was scraped into sterile, degassed chemostat medium, composing the defined community inoculum. The inoculum was thoroughly mixed and used to inoculate three 400-mL Multifors bioreactor system vessels (replicate vessels) already containing 350-mL degassed, sterile chemostat medium at 37 °C, pH 7, and stirring at 50 rpm. Vessels were maintained under anaerobic, batch fermentation conditions for 24 h before switching to continuous fermentation conditions as described earlier. Chemostat medium was fed at a flow rate of 400 mL/day to ensure a 24-h turnover of vessel contents. Vessels were maintained for 21 days, while daily samples (5 × 2 mL) were collected and stored at − 80 °C for subsequent applications.

### DNA extraction

Genomic DNA (gDNA) from fecal samples (100–200 mg) and chemostat cultures (1 mL) were extracted using a Zymo Quick-DNA Fecal/Soil Microbe Kit (Cedarlane Laboratories Ltd., Burlington, ON, Canada) with a modified protocol to enhance lysis of gram-positive cells. Briefly, chemostat cultures were centrifuged at 14,000 rpm for 15 min, and the resulting pellets were resuspended in phosphate buffer and transferred into tubes containing 0.2 g of zirconia beads (supplied by the kit). Samples were disrupted at 3000 rpm for 6 min in Digital Disruptor Genie 3000 (Scientific Industries Inc., Bohemia, NY, USA), followed by 95 °C incubation for 10 min and water bath sonication for 5 min in a Sonicator Ultrasonic Processor XL2020 (Mandel Scientific, Guelph, ON, Canada). Next, samples were treated with 400 μg/mL Recombinant Proteinase K Solution (Ambion, Austin, TX, USA) and heated to 70 °C for 10 min. Samples were allowed to reach room temperature before DNA was extracted following the manufacturer’s instructions. The concentration and quality of gDNA extraction were assessed using a NanoDrop spectrophotometer (Infinite M Nano + , Tecan Group Ltd., Zürich, Switzerland). gDNA samples were stored at − 20 °C until PCR amplification and amplicon sequencing.

### 16S rRNA sequencing and data analysis

We generated amplicon sequence data for variable region three to four (V3–V4) of the bacterial and archaeal 16S rRNA gene following the Illumina protocol for 16S metagenomic sequencing library preparation. To target the V3–V4 region, this protocol used the primers 16S Amplicon PCR forward (5′-TCGTCGGCAGCGTCAGATGTGTATAAGAGACAGCCTACGGGNGGCWGCAG-3′) and 16S amplicon PCR reverse (5′-GTCTCGTGGGCTCGGAGATGTGTATAAGAGACAGGACTACHVGGGTATCTAATCC-3′). Library preparation and high-throughput sequencing were performed at the Mr. DNA Molecular Research LP commercial facility (Shallowater, TX, USA). Sequencing was performed on the Illumina MiSeq platform using the MiSeq Reagent kit V3 (2 × 300 bp paired-end reads). Demultiplexed paired-end reads from Illumina sequencing were clustered into amplicon sequencing variants (ASV) using the DADA2 pipeline (v1.26.0) on R (Callahan et al., 2016). DNA sequence reads were filtered and trimmed based on the quality of the reads for each Illumina run separately, error rates were learned, and sequence variants were determined. The pooling option parameter was set to the pseudo-pooling method unless otherwise indicated. ASV feature tables were merged to combine all information from two separate Illumina runs. Taxonomy classification was assigned using the NCBI 16S rDNA reference database version v5. This resulted in a feature table consisting of 50 samples and 671 ASVs with total reads per sample ranging from 29,140 to 124,045. Taxonomical composition analysis and displays were conducted using the ggplot2 (v3.4.4) package on R. Alluvial plots compare taxonomical composition between samples based on 100% identity at the ASV level and were generated using the ggalluvial (v0.12.5) package in R. Alpha- and beta-diversity analyses were conducted using VEGAN (v2.6–4) package on R. All other statistical analysis was performed using base R.

### Nuclear magnetic resonance spectroscopy

The metabolic output of chemostat communities was assessed using one-dimensional proton nuclear magnetic resonance (^1^H NMR) spectroscopy. Thawed chemostat communities were centrifuged at 14,000 RPM for 15 min to remove microbial cells, followed by filtration of sample supernatant through sterile 0.22-μm polyethersulfone (PES) filters (GE Whatman, Mississauga, ON, Canada). Filtered supernatants were spiked-in with an internal standard, 99.9% D_2_O with 5-mM 4,4-dimethyl-4-silapentane-1-sulfonic acid (DSS), and 0.2% sodium azide to a final concentration of 0.5-mM DSS. Samples were stored in 5-mm glass NMR tubes (535-PP-7, Wilmad, Vineland, NJ, USA) at 4 °C until scanning in a Bruker Avance 600 MHz NMR spectrometer (NMR Centre, AAC, University of Guelph, Guelph, ON, Canada) as previously described by Ganobis et al. [48]*.* Briefly, spectra were acquired using the first increment of a 1D ^1^H NOESY pulse sequence with tmix of 100 ms, 4-s acquisition time, 1-s relaxation delay, and a spectral width of 12 ppm. Sample pH was measured using pH colorimetric strips (GE Whatman, Mississauga, ON, Canada) within 1 day of scanning. Chenomx NMR Suite 7.0–7.7 (Chenomx Inc., Edmonton, AL, Canada) software was used to process spectra and subsequently identify and quantify metabolites. Phase and baseline corrections were made manually, while shim and chemical shape corrections were performed automatically by the software. Metabolites were identified by matching the peaks on the spectra with projections for predicted metabolites in the software’s 600-MHz compound library. Metabolites were quantified from the area of the projected signal by using the known concentration of DSS and the area of the DSS peak. Variation of the concentration of NMR-profiled metabolites over time was displayed using ggplot2 (v3.4.4) package on R.

### Mass spectrometry

The metabolic output of chemostat communities was also assessed using untargeted hydrophilic interaction chromatography coupled with mass spectrometry (HILIC-MS, negative mode) Metabolomics data were acquired at the Calgary Metabolomics Research Facility (CMRF, https://calgarymetabolomics.org/services), supported by the International Microbiome Centre and the Canada Foundation for Innovation (CFI-JELF 34986). Metabolites were identified using a library of 450 small molecule standards that were associated with microbial metabolism (Suppl. Table 3). Briefly, aliquots of 2-mL frozen chemostat cultures were thawed on ice and centrifuged at 18,213 g for 15 min to remove microbial cells. An equal volume of HPLC-grade methanol (Millipore-Sigma, Catalog no. 34860) was added into the supernatants of each sample, followed by incubation on ice for 30 min, vortexing each sample every 10 min to ensure proper liquid–liquid extraction. Next, samples were centrifuged at maximum speed (18,213 g) for 10 min, and the extracted organic layer was recovered and kept at − 80 °C overnight. Samples were subsequently thawed on ice and centrifuged at maximum speed (18,213 g) for 10 min. Sample supernatant was diluted 1:50 using pre-chilled 50% HPLC-grade methanol and shipped on dry ice to CMRF for HILIC-MS analysis. Chromatographic separation of the extracted metabolites was achieved on a Syncronis HILIC UHPLC column (2.1 mm × 100 mm × 1.7 µm, Thermo Fisher) using a binary solvent system at a flow rate of 600 µL/min" solvent A, 20-mM ammonium formate pH 3.0 in mass spectrometry grade H_2_O; solvent B, mass spectrometry grade acetonitrile with 0.1% formic acid (%v/v). Subsequent mass spectrometry analysis was performed using a Q Exactive™ HF Hybrid Quadrupole Orbitrap™ Mass Spectrometer (Thermo Fisher) coupled to a Vanquish™ UHPLC system (Thermo Fisher). Chromatographic separation A sample injection volume of 2 µL was used. The mass spectrometer was run in negative full scan mode at a resolution of 240,000, scanning from 50 to 750 mass-to-charge ratio (m/z). Retention time (rt) and m/z ratio of peaks identified in the experiment were annotated by comparison to known rt, and m/z values that correspond to a commercial library of 350 metabolic standards and their respective abundances (measured in arbitrary units (AU)) were derived from the area under the curve (AUC) using El-Maven. Individual metabolites that were consistently above noise (in AU) were compared relatively across samples after normalization to their corresponding baseline value identified in bacteria-free chemostat medium and subsequent log_10_ transformation. Variation of each HILIC-MS-profiled metabolite over time was normalized to fold change from day 2 of culture and displayed as heatmaps using the ComplexHeatmap (v2.14.0) package on R.

### Metabolite-bacteria correlation analysis

Covariation between bacterial metabolites and ASV relative abundances data is presented in the form of a heatmap diagram based on Spearman’s correlation coefficients that are calculated based on Euclidean distances for each microbe-metabolite pair (NMR using mM in Supp. Table 4, mass spectrometry using arbitrary units in Supp. Table 5). The correlation coefficients were color-coded by the intensity of the Spearman correlation coefficient to show the strength of the correlative relationship over the trajectory of longitudinal chemostat culture. Heatmaps of correlation analysis were conducted using the ComplexHeatmap (v2.14.0) package on R. Scatter plots were generated using the ggplot2 (v3.4.4) package in R.

## Results

### Chemostat-cultured fecal samples from young children generate communities representative of the donor microbiome

**Fig. 1:**
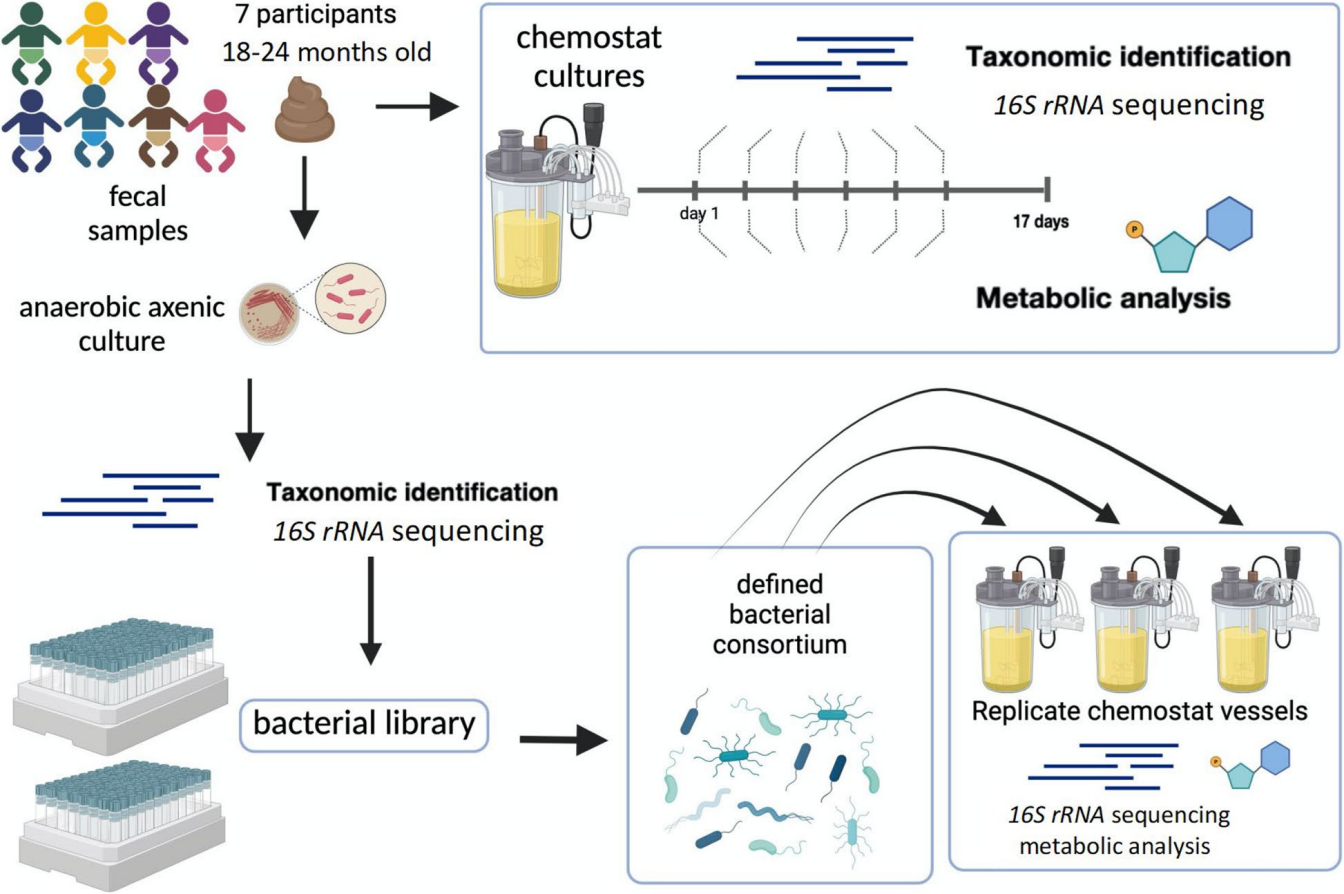
Infographic: fecal samples isolated from seven, 18–24-month-old study participants were selected for study. The fecal samples were used to inoculate individual continuous-flow bioreactors (chemostats) designed to emulate conditions of the human distal intestine. These cultures were sampled longitudinally and examined for bacterial composition by 16S *rRNA* sequencing. The resulting amplified sequence variants (ASV) were compared to the original fecal samples to assess the representativeness of the chemostat model of early childhood fecal communities. In parallel, day 1 and day 28 chemostat culture samples were used to inoculate multiple types of plated media from which axenic isolates were isolated and subjected to 16S *rRNA* sequencing and taxonomic identification. These isolates were archived to create a curated library of early childhood fecal bacterial strains. A defined, complex consortium was prepared from the library and used to inoculate replicate chemostat vessels to assess reproducibility of the model. Samples from replicate vessels were withdrawn over time for 16 s *rRNA* sequencing to evaluate taxonomic composition and for metabolomic analysis of bacterial output in a host-free setting

Fecal samples were isolated from seven, 18–24-month-old, healthy children. The sample donors were members of the longitudinal DIABIMMUNE birth cohort that followed infants from birth to 3 years with a comprehensive collection of environmental and clinical metadata [16, 21]. At the time of sampling, the children were consuming solid foods and had not been exposed to antibiotics. The selected samples displayed beta-diversity dissimilarity indices of taxonomic composition representative of the mean for the cohort group (Bray–Curtis indices selected sample/group median: sample NS0: 0.81/0.82, sample NS1: 0.79/0.82, sample S2: 0.69/0.69, sample S3: 0.7/0.69, sample NS4: 0.83/0.82, sample S5: 0.65/0.69, sample NS6: 0.84/0.82) [16, 21]. The seven fecal samples were used to inoculate individual continuous-flow bioreactors (chemostats) designed to emulate conditions of the human distal intestine (Fig. 1). The taxonomic composition of the selected *ex* vivo fecal and fecal-derived chemostat cultured samples was identified by *16S rRNA* V3-V4 gene region sequencing (Fig. 1). The data were analyzed with the DADA2 (v1.26.0) software package using a pseudo-pooling inference method (Suppl. Figure 1).

**Fig. 2:**
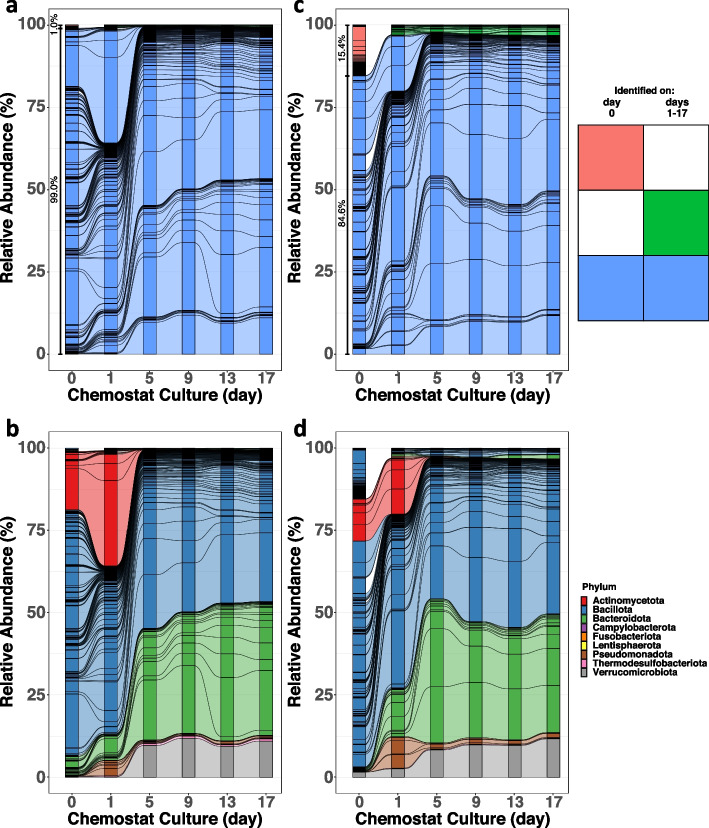
Participant fecal sample cultured in the chemostat model generates a bacterial community representative of the donor’s microbiome. Relative abundances of amplicon sequence variants (ASV) that represented bacterial composition were identified by Illumina MiSeq *16S rRNA* gene sequencing of fecal samples and of longitudinal chemostat communities cultured from two healthy participants. **a**, **b**, **c**, **d** Alluvial plots show the relative abundance of ASVs identified on the fecal sample (day 0) and the chemostat cultures inoculated from NS0 (**a**, **b**) and NS1 (**c**, **d**) individuals, taken over time. ASVs, indicated by colors, are stratified by their identification on the indicated days of culture in the key (upper right). The relative abundance of ASVs from the fecal sample found in the chemostat culture is indicated in brackets adjacent to the day 0 bar. **b**, **d** Colors represent the nine predominant phyla. ASVs are ordered by prevalence categories

Fecal samples were cultured separately in a single-stage continuous-flow chemostat system that simulates conditions in the human distal colon and supports the growth of adult human microbiota [25, 47]. Temperature, pH, medium feed rate, stirring, and anaerobic conditions are tightly controlled in the model [26]. After inoculation with the fecal sample, batch fermentation was carried out for 48 h, followed by continuous culture in which fresh medium was fed to the vessels at a rate that matched medium removal and was set to emulate a colonic transit time of 24 h. A flow rate of 400 mL/day ensures a 24-h turnover of vessel contents. During continuous culture, the bacterial composition of the fecal-derived communities was serially profiled by *16S* rRNA gene sequencing. Bacterial taxa identified in the early childhood fecal sample (chemostat inoculum) and their respective fecal-derived chemostat culture over time were compared to assess the capacity of the model to support the donor’s microbiome (Fig. 2; Suppl. Figure 2). Over the time of culture, the bacterial composition of NS0 and NS1 chemostats displayed 99% and 84.6% of the amplicon sequencing variants (ASVs) identified in their respective fecal inoculum (Fig. 2a and c; in blue). A minority of ASVs identified in the original fecal samples were not observed in their derivative chemostat cultures (Fig. 2a and c colored red). In the NS1 culture, 4% of ASVs identified across days 1–17 of chemostat culture were not identified in the original fecal samples suggesting they were of low abundance and had adapted to growth in culture (Fig. 2c colored green).

Within the NS0 sample, the majority of ASVs identified in the fecal sample inoculum (day 0) were also identified in the chemostat culture (Fig. 2a). The vast majority of fecal ASVs (96.95%) were present at all culture time points. A minor fraction of ASVs was identified only on day 0 or on days 1–17 (Fig. 2a). Less than 1% of ASV identified in the fecal inoculum were absent from all time points in the chemostat culture. In agreement with these findings, bacterial alpha-diversity metrics in the NS0 fecal sample were similar to those of the NS0 chemostat culture (in fecal sample: Shannon 3.78/Chao-1 305.5; at day 17 of culture: Shannon 3.11/Chao-1 267.2). The chemostat-cultured NS0 community succession and stabilization were accompanied by an expanded proportion of Bacteroidota and Verrucomicrobiota and contraction of Actinomycetota phylum members by 5 days of culture (Fig. 2b). For the NS1 sample, 84.6% of the ASVs identified in the fecal sample (day 0) were also identified in the fecal-derived culture (Fig. 2c colored blue). The remaining minority of fecal ASVs were not identified at any point in culture, representing uncultured bacteria. At culture day 5, the NS0-derived community represented 99%, and the NS1-derived community displayed 84.6% of the relative abundance of their respective original fecal sample (Fig. 2a and c). Most of fecal bacteria absent from the NS1-derived cultures were Bacillota and Actinomycetota (Fig. 2d). Many of the ASVs absent from the NS1 fecal-derived cultures were identified in the cultured NS0 community, suggesting that the chemostat could support their growth in the context of a different microbial consortium (Suppl. Table 6).

To determine how the growth trajectories observed for NS0 and NS1 were representative of fecal microbiota of 18–24-month-old donors, samples from five additional DIABIMMUNE participants (NS3, NS6, S2, S3, S5) were analyzed in chemostat cultures. In these five samples, 64.4 to 93.4% of the ASVs identified in the fecal inoculum were also observed in the derivative cultures (Suppl. Figure 2a, b, c, d, e; colored blue). Compared to the original fecal inoculum, inter-individual variation in ASV abundance and representation was observed in chemostat cultures from the seven donors. Bacillota was the most abundant phylum in several samples followed by the Actinomycetota, Bacteroidota, and Verrucomicrobiota (Fig. 2b, d; Suppl. Figure 2f, j). A Bray–Curtis dissimilarity principal coordinates analysis (PCoA) of all seven donors demonstrated that at day 17, the cultures represented, and were most like, the composition of the donor’s fecal community (Suppl. Figure 2 k). To examine the trajectories of the chemostat communities at the family level, ASV relative abundances for the seven samples were collapsed according to their highest-likelihood family-level classification level (Suppl. Tables 7, 8, 9, 10, 11, 12, 13) with each family stratum represented by a separate color (Suppl. Figure 3). Taken together, longitudinal analysis of these seven early childhood donors suggested that chemostat model supported growth of most taxa observed in their fecal samples and retained donor-specific features of the microbiota.

### Isolation of bacterial strains from fecal-derived chemostat community

**Fig. 3:**
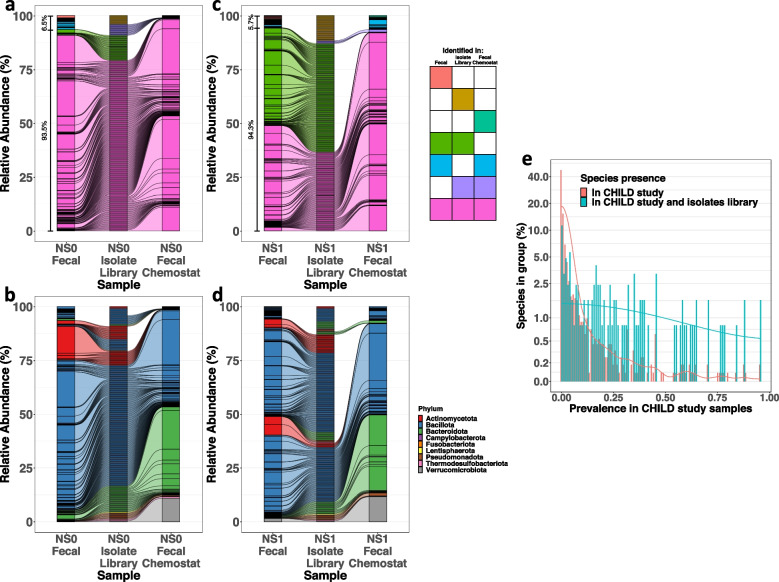
A library of bacterial strains isolated from the fecal-derived chemostat community is representative of the donor’s microbiota. Bacterial strains from the fecal-derived chemostat community of NS0 or NS1 study participants were isolated by axenic culture using a variety of media formulations. Eighty unique bacterial strains were archived, constituting a library of the NS0 donor, and 118 unique bacterial strains were archived, constituting a library of NS1 donor. **a**, **b**, **c**, **d** Alluvial plots show relative abundances of bacterial ASVs identified in the fecal sample (left columns), fecal-derived chemostat community at stability (right columns), and the respective bacterial isolate library (idealized equal proportions of ASVs shown for comparison, middle columns) from NS0 (**a**, **b**) and NS1 (**c**, **d**) individuals. Lines connecting the columns indicate identical ASV across samples. **a**, **c** ASVs were stratified by the presence or absence in each sample, indicated by color. The relative abundance of ASVs from the fecal sample found in the isolate library is indicated in brackets adjacent to the left fecal sample bar. **b**, **d** Colors represent the nine predominant phyla. ASVs are ordered by prevalence categories. **e** Prevalence distribution of all CHILD cohort infants at 12 months of age (*n* = 842 samples) bacterial species (red) or species matched to bacterial isolates from NS0 and NS1 libraries (blue) displaying extensive similarity in representation of bacteria between the two data sets across both common and rare prevalence species

The chemostat model supported growth of a large portion of bacterial taxa identified in these early childhood fecal samples. We leveraged this opportunity to isolate and archive bacterial strains representative of 18–24-month-old children. Axenic isolation of bacteria was performed with an array of culture media (Suppl. Table 2). We generated a taxonomically diverse library of NS0 and NS1 donor-derived bacterial strains isolated from a 24-h batch fermentation of the fecal samples and also from bacteria present at day 28 of chemostat cultures (Suppl. Figure 4). These efforts resulted in isolation of 80 NS0-derived and 118 NS1-derived unique bacterial strains (Fig. 3; Suppl. Figure 4, Suppl. Tables 14, 15). Based upon 16S *rRNA* gene sequence analysis of the isolates, the NS0 library captured 93.5% and the NS1 library 94.34% of the ASV identified in the corresponding fecal sample. These isolates included ASVs that became undetectable in the fecal-derived chemostat culture over time, indicating that these strains were viable in the original fecal sample. Strain isolation from both day 1 fermentation and established chemostat cultures successfully captured a vast majority of the fecal identified ASV (Fig. 3a and c). Less than 6% of the NS1 and less than 7% NS0 fecal ASVs were not isolated by either method (Fig. 3a and c). Thus, the chemostat model enabled cultivation of 18–24-month-old participant’s fecal bacteria.

To compare the diversity and richness of NS0 and NS1 bacterial isolates with the gut bacteria from other young children, we analyzed *16S rRNA* sequence data from 842 fecal samples isolated from 12-month-old participants in the CHILD cohort study [15, 49]. As expected from inter-individual variation, the prevalence distribution of species in CHILD samples shows that the majority were “private” species found in one or a few individuals, while a minority were “common” species to many individuals (Fig. 3e). The NS0 and NS1 isolate libraries contained 124/1246 (9.9%) of the species identified in the *n* = 842 CHILD participants, including an equal distribution of the “private” as well as common species in this cohort (Fig. 3e). Collectively, these results indicate that bacterial communities supported by the chemostat culture platform and isolated by axenic methods were representative of the bacterial taxa identified in early childhood fecal microbiota.

### Chemostat cultures of defined bacterial consortia generate a reproducible community

To evaluate the reproducibility of the chemostat model, three vessels were inoculated with the identical 118-strain NS1 library mixture, and the taxonomic composition for the triplicate vessels was followed by longitudinal 16S *rRNA* sequence analysis. We prepared individual monocultures of the 118 strains and combined them to generate a consortium used to inoculate fresh chemostat vessels in triplicate. In each of the three vessels, the 118-strain inoculum achieved stable ASV abundances from 5 days of culture onwards (Suppl. Figure 5a, b, d, e, f). The chemostat-cultured bacterial consortia were representative of the original inoculum (day 0) with 95.5–99.7% of fecal ASV abundance identified in the chemostat culture. We observed the loss of ~ 3% of the inoculum relative abundance after day 1 of culture indicating that a subset of strains was excluded from the chemostat community (Suppl. Figure 5a, c, e). The trajectory of this defined bacterial consortium mirrored that observed in the fecal-derived community, including expansion of the Bacteroidota and Verrucomicrobiota phyla with concurrent contraction of members of the Actinomycetota phylum (Suppl. Figure 5b, d, f).

**Fig. 4:**
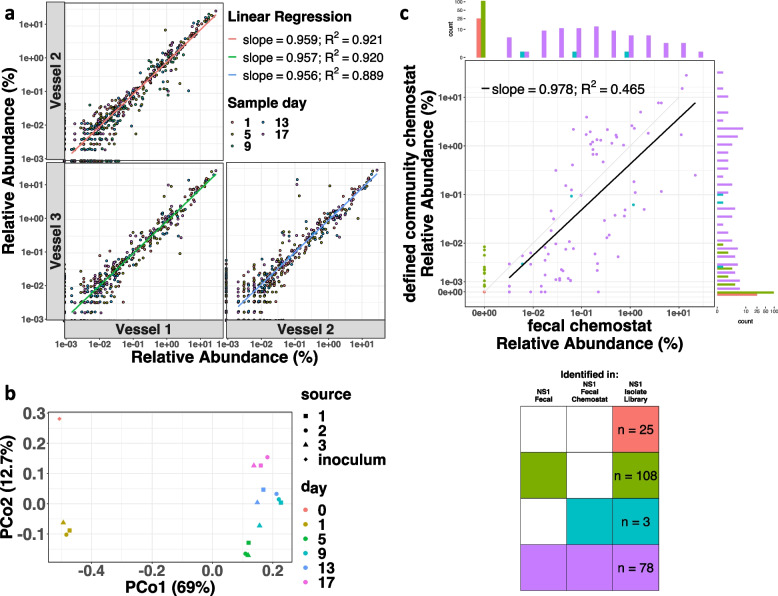
Isolated bacterial strains generate a reproducible bacterial community. Triplicate chemostat vessels were inoculated with a defined consortium of all 118 strains from the NS1 bacterial library. **a** Pair-wise comparison of ASV relative abundances identified in each replicate vessel of the defined chemostat community across days sampled. Linear regression analysis and coefficients are shown. **b** Bray–Curtis dissimilarity PCoA of three replicate vessels of defined communities over time based on ASV relative abundances. The first and second principal coordinates are shown. Values in the brackets indicate the % of total variability explained by each principal coordinate. Three different point shapes distinguish the replicates, and point color indicates the day of culture. **c** Pair-wise comparison of each ASV relative abundance in the fecal-derived community versus the defined community at stability (represented by day 17 of culture in the chemostat). ASVs are colored by prevalence category. Histograms show the frequency distribution of each prevalence category in each community. Key (lower right) indicates numbers of strains identified in isolate library and their identification in original fecal and fecal-derived chemostat

The relative abundances of each of the ASV were well correlated between the three vessels (Fig. 4a, R^2^ = 0.89–0.92) indicating that this chemostat model provided a reproducible culture platform for complex communities composed of gut bacteria from 18–24-month-old children. Bray–Curtis dissimilarity PCoA analysis of the bacterial composition in the triplicate vessels revealed tight clustering between the vessels on each day of culture indicating a high degree of reproducibility (Fig. 4b). Consistent with our observations for fecal-derived community behavior, bacterial composition shifts were observed between days 1 and 5 of culture and reached relative compositional stability across the vessels at day 5 (Suppl. Figure 5). We compared the ASV relative abundance of the 118-strain defined community (at culture day 17) with the same parameter in the original NS1 fecal chemostat community. The two were moderately correlated with the least correlated ASV present at low abundances (Fig. 4c; ^−^slope = 0.978; *R*^2^ = 0.465). These data demonstrate that the same taxa that were lost from the NS1 fecal-derived chemostat community competed poorly in the NS1 118-isolate defined consortium cultures indicating that similar bacterial communities were generated from both sources (Fig. 4c; green and red bars).

### Identification of microbe-derived metabolites from early childhood bacterial communities

Bacterial metabolites mediate many interactions between the human host and the gut microbiota. Since these metabolites can be produced, chemically modified and utilized by human cells and intestinal microbes, it is challenging to define the cellular origins of the fecal-derived metabolites. The chemostat models a host-free, nutritionally defined system enabling investigation of the early childhood gut microbial metabolism. We examined metabolites in the chemostat cultures over time using complementary approaches: proton nuclear magnetic resonance (^1^H NMR) and high-performance liquid chromatography tandem-mass spectrometry (HILIC-MS). ^1^H NMR provides absolute metabolite quantification for a limited number of abundant metabolites. HILIC-MS metabolomics affords relative quantitation and greater sensitivity for a larger pool of analytes (*n* = 370, Suppl. Table 3).

**Fig. 5:**
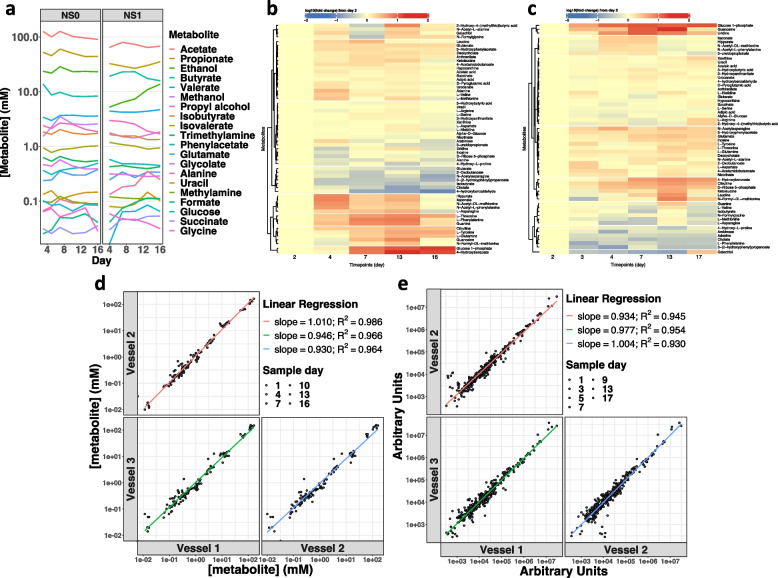
Microbial metabolites produced by gut bacterial communities display reproducible trajectories. **a**, **b**, **c** Longitudinal trajectories of metabolites were identified in early childhood gut bacterial communities cultured in the chemostat model. **a** Longitudinal quantification of metabolites in NS0 and NS1 fecal-derived chemostat cultures analyzed by ^1^H NMR. Each metabolite is distinguished by color and concentration displayed over time. Metabolites in the legend key are ordered by their concentration on day 17 of NS0 culture. **b**, **c** Heatmap and hierarchical clustering analysis of HILIC-MS identified metabolite levels over time in NS0 (**b**) and NS1 (**c**) fecal-derived communities. Levels of each metabolite were normalized by fold change relative to day 2 of culture and illustrated by the color gradient. **d**, **e** Pair-wise comparison of microbial metabolites identified in each replicate vessel of the defined consortium cultured in the chemostat inoculated with the NS1 bacterial library across days sampled. Linear regression analysis and coefficients are shown

We used ^1^H NMR to identify 20 metabolites in the NS0 and NS1 fecal-derived chemostat cultures (Fig. 5a). The composition and abundances of these metabolites were characteristic of cultured human gut bacterial communities [50–52] and displayed stable concentrations in the chemostat from day 8 onward (Fig. 5a). The principal metabolite classes observed were fatty acids, amino acids and their derivatives, primary alcohols, and amines (Suppl. Table 16). In the HILIC-MS analysis, more than 60 metabolites were identified longitudinally in the NS0 (Fig. 5b) and NS1 (Fig. 5c) fecal-derived chemostat cultures. These analytes included amino acids and their derivatives (*n* = 28), fatty acids and their conjugates (*n* = 9), monosaccharides and disaccharides (*n* = 7), purines (*n* = 6), and pyrimidines (*n* = 6). Five metabolites were detected using both methods, supporting the utility of multiple approaches to profile microbial metabolites (Suppl. Table 16). For the 20 analytes identified by ^1^H NMR, the coefficient of variation (CV) between day 2 and day 17 of culture was modest (median CV 14.31% and 13.15% for NS0 and NS1 fecal-derived communities, respectively), reflecting the production of abundant metabolites by a relatively stable bacterial community, in a stable nutrient environment.

Similarly, many HILIC-MS-profiled metabolites showed stable abundances from day 7 of culture (Fig. 5b, c). The NS0 and NS1 fecal-derived cultures shared most metabolites detected by both analytical methods. However, each community exhibited unique trajectories (Fig. 5a, b, c) consistent with distinct compositional dynamics of the early life fecal samples in this study (Fig. 2; Suppl. Figure 2). Greater variation between days 2 and 16 of culture was observed in HILIC-MS-detected metabolites (median CV 48.7% and 44.3% for NS0 and NS1 fecal-derived communities, respectively) consistent with the higher sensitivity of this method for lower abundance analytes.

We next evaluated the metabolites produced in the communities cultured from the NS1 bacterial 118-strain library. Twenty bacterial-derived metabolites were identified and quantified in the three replicate vessels by ^1^H NMR, including short chain fatty acids, amines, alcohols, and organic acids (Suppl. Table 17). Their composition, abundance, and trajectory were highly reproducible between replicate vessels, and most metabolite concentrations were stable by culture day 9 (*R*^2^ > 0.97 Fig. 5d; *R*^2^ ≥ 0.93 Fig. 5e). In the HILIC-MS analysis, 57 bacterial-derived metabolites were identified in the defined communities, including amino acids and derivatives, acids and conjugates, purines, and monosaccharides and disaccharides (Suppl. Table 17). Most of the identified metabolites achieved relative stability by day 9 of culture.

### Association between bacterial composition and metabolic output

The chemostat model provides the opportunity for longitudinal, contemporaneous sampling of taxonomic composition and metabolic output. To investigate associations between bacterial taxa and metabolites in the NS1-118 strain defined consortia, we performed correlation analyses on the abundances of ASVs and metabolites from day 2 of culture in three replicate vessels. ASV-metabolite Pearson correlation coefficients from ^1^H NMR (Suppl. Figure 6a) and HILIC-MS (Suppl. Figure 6b and c) datasets were normalized using z-score. Eighteen significant ASV-metabolite correlations were identified in these cultures (Pearson, *FDR* < 0.086, Suppl. Tables 4 and 5). Examples of significant correlations were correlations of acetate concentration with *Bacteroides stercoris* (positive) and with *Bacteroides uniformis* (negative) abundances in the NS1-defined communities (Suppl. Figure 6b, c). *B.* *stercoris* abundance was also associated with succinate (slope = 0.379, Pearson cor. coefficient (R) = 0.803, *R*^2^ = 0.646) and glutarate levels (slope = − 0.105, Pearson cor. coefficient (R) = − 0.918, *R*^2^ = 0.843).

Taken together, these studies report on the utility of a continuous-flow chemostat system designed to mimic the human distal colon to propagate microbial communities derived from healthy early childhood stool. These chemostat cultures were representative of the bacterial diversity in early childhood fecal samples and produced reproducible compositionally and metabolically reproducible communities. These chemostat cultured communities recapitulated multiple aspects of the early childhood fecal ecosystem and enabled the investigation of relationships between bacterial strains and metabolic functions and the isolation of a complex libraries of bacterial strains. Our data suggest that this chemostat model, combined with high-throughput sequencing and axenic bacterial isolation, enables a comprehensive, integrated approach to study gut microbiota composition and function during the first 18–24 months of life.

## Discussion

Multiple in vitro culture models have been developed and employed for the study of the human gut microbiota [24, 25, 27–38, 47, 53, 54]. Continuous-flow chemostat systems have shown to be useful for simulating adult microbial communities, although few have been applied to the early childhood gut microbiota [27, 34, 35, 39–42, 55, 56]. Here, we have characterized seven early childhood gut bacterial communities cultured in a continuous-flow, single-stage chemostat culture “Robogut” model of the human distal colon [47] to advance experimental systems applicable to understanding the early life gut microbiota.

To demonstrate the efficiency of this chemostat model to emulate the early life gut microbiota, our study focuses on three key points: (1) *generalizability* of the data for early childhood gut microbiota, (2) taxonomic *representativeness* of organisms supported by the chemostat model, and (3) *reproducibility* capacity of the model to produce similar communities from the same inoculum. Our study showed that chemostat-cultured communities derived from seven fecal samples retained a large proportion of the bacterial diversity present in the original inoculum, thus efficiently representing the donor’s microbiota. The representativeness of the chemostat communities of their original fecal microbiota is supported by identification of a majority of fecal-identified ASV in the derivative chemostat cultures (mean = 86.7%, range 64.4–99%). Variability in the proportion of bacterial taxa in the chemostat cultures decreased over time, demonstrating relative community stabilization by 5 days of culture. This model successfully cultured the bacterial phyla that constitute the human gut microbiome, including Bacillota, Bacteroidota, Verrucomicrobiota, and Pseudomonadota, while demonstrating lower success in supporting the growth of Actinomycetota. Examples of bacteria successfully captured in this model include species of *Ruminococcus*, *Akkermansia*, *Alistipes*, and *Bacteroides* genera. Less than 2% of ASVs identified in the NS0 fecal sample failed to persist in chemostat culture, while nearly a third of NS1 fecal ASVs were not identified after day 1 of culture. Examples of bacteria poorly supported in the NS1 fecal-derived communities included species of *Blautia* and *Hungatella* genera that were present in NS0 cultures indicating that they can be supported in the chemostat model where they are affected by other community members. One explanation for these findings is that the NS1 cultured community failed to support the growth of these species due to a lack *extrinsic factors* such as nutrients, metabolites, or niches. Another possibility is that the NS1 bacterial strains had distinct intrinsic requirements from strains of the same species in the NS0 community. Such *strain-specific differences* could result in host factor dependencies which were not recapitulated in culture. Distinctions between these early childhood fecal-derived cultures suggest that biological differences present in the original fecal bacterial communities were preserved in the chemostat model. Future studies can be designed to test both intrinsic and host-associated extrinsic factors that influence microbial community structure.

In addition to the utility of this chemostat model to study the dynamics of complex gut bacterial communities, we deployed this system to integrate community-level with strain-level analyses. We took advantage of the ability of the chemostat model to propagate diverse fecal bacteria from small amounts of stool to isolate and archive bacterial strains. Axenic isolation retrieved 80-118 unique bacterial isolates representing > 94% of the fecal ASV abundance of these early childhood samples. Isolated bacterial taxa included organisms that were not identified in the original sample by 16S *rRNA* gene sequencing likely due to their low fecal abundance. The resulting early life gut bacterial repertoire is a well-characterized, representative, and renewable resource for the study of microbial community dynamics, as well as potential physiological effects using in vivo colonization of mouse models [57].

The strains from the archived library were cultured in the chemostat model resulting in communities assembled from defined bacterial isolates. Construction of defined communities from curated libraries permits community-level genomic and metabolomic analysis of bacterial strains under reproducible conditions, as evidenced by similar taxonomic and metabolic trajectories we observed in replicate cultures [58]. The biological impact of such defined microbial consortia can be probed by their inoculation into animal models or inclusion in tissue organoid models. Our results indicate that the chemostat model is useful for analyzing microbial communities associated with human diseases or responses to therapeutics by demonstrating reproducibility and maintenance of community-level dynamics that are challenging in settings of fecal microbial transfer (FMT) [59] and mono-colonization experiments.

A fertile area of investigation is the metabolic capacities of complex human gut bacterial communities. Confounding factors, including microbial cell viability, community dynamics, host interactions, and nutrient availability, modulate the metabolic activity of community members in the context of the environment. Here, we used analytical chemistry methods to identify microbial metabolites in fecal-derived chemostat cultures [50, 52, 60–62]. Analytes representing diverse branches of microbial metabolism were identified, bile acids and their derivatives [63, 64], carbohydrate fermentation to alcohols [65, 66], short-chain fatty acids [67–72], and amino acid metabolites that can generate branched-chain fatty acids and indole derivatives [73–75]. Metabolomic analysis of chemostat cultures identified active metabolism of microbial communities independent of host factors [76]. Moreover, longitudinal co-analysis of metabolites and bacterial taxa in the chemostat system enables prediction of active microbe-metabolite relationships within communities.

A limitation of the chemostat model under the conditions selected in this study is the lack of support for specific bacterial taxa. Multiple studies using bioreactor platforms have reported a decline in taxonomic richness following the inoculation of stool samples [27, 34, 47, 77], including lower frequencies of Actinomycetota in fecal-derived communities from toddlers [41, 56]. One explanation for these observations is that the growth of particular bacteria depends on specific niches of the host environment [35, 36, 78, 79]. A single-stage chemostat with a homogenous media is unable to mimic the complex biogeographic and chemical conditions of the human gut. Supplementation of the basal media with lactose, human milk oligosaccharides, or iron depletion has been reported to enhance growth of Actinomycetota in early childhood gut microbiota in bioreactors [41, 56], providing potential approaches to enhance the value of these models in the context of specific research questions.

## Conclusions

In summary, we present a methodological and analytical framework to assess community-level dynamics of bacterial taxonomy and small molecule metabolomics applicable to early childhood gut microbiota. The microbial communities propagated in the chemostat model were reproducible and representative of the original stool inoculum. These communities created an abundant source to create libraries of bacterial strains, thus integrating community-level with strain-level investigation. To our knowledge, this is the first in-depth characterization of the longitudinal dynamics of cultured infant gut bacterial communities using a simulator model of the human colon and provides a framework for multi- “omic,” functional analyses of infant and early childhood gut microbiota.

## Supplementary Information

Supplementary Material 1. 

Supplementary Material 2: Figure 1. Relative abundance of ASVs identified in early childhood fecal sample and fecal-derived chemostat culture using DADA2 non-pooled or pseudo-pooled inference methods. DADA2 method aims to accurately reconstruct the exact amplicon sequence variants (ASVs) truly present in a sample from the noisy amplicon sequencing reads [80]. By default (non-pooled method), DADA2 parameters are set to achieve high accuracy by reducing the number of spurious ASV outputs and increasing the specificity. However, the tradeoff for that high specificity is that sensitivity, particularly to rare variants, is reduced by the default non-pooled method. The pseudo-pooled sample inference method allows information to be shared across related samples in a dataset and is particularly effective in longitudinal and inoculation experiments in which samples are taken repeatedly from the same source. This method is expected to improve sensitivity and provide a more accurate description of ASVs at very low frequencies without demanding high computational time. The alternative full pooled sample inference method explicitly infers ASVs across the dataset. This method is also expected to improve sensitivity and provide a more accurate description of ASVs at very low frequencies, at a cost of higher computational time which extends proportionally as a function of the number of samples squared. Here we compare the total number and relative abundance of ASVs identified in two early childhood fecal samples and fecal-derived chemostat cultures using DADA2 non-pooled, pseudo-pooled or full-pooled inference methods. We observe that the pseudo-pooled inference method increased the number of ASVs identified on fecal samples and fecal-derived chemostat cultures. Pseudo-pooling of NS0 samples allowed the identification of additional 81 – 89 ASVs, while in NS1 samples, we observed 41 – 80 new ASVs identified. As expected, most new variants discovered by this method are present in low abundance. In contrast, the pseudo-pooled method does not affect the presence and relative proportion of high abundant ASVs, suggesting high specificity. Full-pooling of NS0 samples allowed identification of an additional 19 – 31 ASVs, while in NS1 samples, this approach returned a loss of 85 ASVs and a gain of 28 new ASVs identified. As expected, most variation in ASVs discovered by these methods were present in low abundance. These results indicate that the pseudo-pooled method increased DADA2 sensitivity, preserved specificity, and allowed identification of rare variants in both fecal samples and fecal-derived chemostat cultures. We observed no clear benefit to the full pooling inference method that was proportional to the increased computational time it required. All figures in this manuscript, except this one, have been generated using the pseudo-pooled inference method.

Supplementary Material 3: Figure 2. Five additional fecal samples cultured in the chemostat model generate bacterial communities representative of the donor’s microbiome.Relative abundances of amplicon sequence variants (ASV) that represent bacterial composition were identified by Illumina MiSeq 16S rRNA gene sequencing from fecal samples and longitudinal chemostat communities of an additional five healthy 18-24-month-old participants. (a‑j) Alluvial plots show the relative abundance of ASVs identified on the fecal sample (day 0) and the chemostat culture over time from S2 (a, f), S3 (b, g), NS4 (c, h), S5 (d, i) and NS6 (e, j) individuals. (a-e) ASVs, indicated by colors, are stratified by presence or absence in on the indicated days of culture. The relative abundance of ASVs from the fecal sample found in the chemostat culture is indicated in brackets adjacent to the day 0 bar. (f-j) Colors represent the nine predominant phyla. ASVs are ordered by prevalence categories. (k) Bray-Curtis dissimilarity PCoA of (day 0) fecal sample and day 17 chemostat samples from seven study participants. The first and second principal coordinates are shown. Values in the brackets indicate the % of total variability explained by each principal coordinate. Point shape indicates sample type, and point color indicates the source individual.

Supplementary Material 4: Figure 3. Family-level collapsed bacterial communities for fecal and fecal-derived chemostats of all seven fecal samples: Relative abundances of amplicon sequence variants (ASV) that represent bacterial composition were identified by Illumina MiSeq 16S rRNA gene sequencing from fecal samples and longitudinal chemostat communities of seven healthy 18-24-month-old participants. ASV relative abundances were collapsed according to highest-likelihood family-level taxanomic classification via BLAST. Alluvial plots show the relative abundance of bacterial families identified on the fecal sample (day 0) and the chemostat culture over time from NS0 (a) and NS1 (b), S2 (c), S3 (d), NS4 (e), S5 (f) and NS6 (g) individuals. Colors represent bacterial families; families are ordered alphabetically by phyla name, then family name. Families appear in each corresponding legend in the same order as in the plot. Families with <1% relative abundance in the samples shown are plotted in grey.

Supplementary Material 5: Figure 4. A library of early childhood gut bacterial isolates. Bacterial strains from the fecal-derived chemostat community of NS0 or NS1 individuals were isolated by axenic culture using a variety of media formulations (Suppl. Table 2 ). 80 unique bacterial strains were archived, constituting a library of NS0 sample (Suppl. Table 14 ), and 118 unique bacterial strains were archived, constituting a library of NS1 sample (Suppl. Table 15). (a) Phylogenetic tree of all gut bacterial isolates (species-level) identified by 16S rRNA gene sequencing of NS0 and NS1 libraries. Colored squares indicate phylum. (b) The relative abundance distribution of each isolate species in the fecal samples of CHILD cohort infants at 12 months old is shown (*n*=842 samples). 16S rRNA gene sequencing dataset from CHILD cohort was obtained at the Sequence Read Archive of NCBI via accession number PRJNA657821.

Supplementary Material 6: Figure 5. A library of isolated bacterial strains generates a reproducible bacterial community. (a, c, e) Alluvial plots show the relative abundance of ASVs from the 118 strains defined inoculum (day 0) and following inoculation of three replicate chemostat vessels, sampled over time (key upper right). (b,d,f) ASVs in the inoculum and triplicate cultures stratified by their identification in the culture over time, where colors represent the nine predominant phyla. ASVs are ordered by prevalence categories.

Supplementary Material 7: Figure 6. Association of bacterial taxa and metabolites in replicate chemostat cultures. Correlation analysis of bacterial composition and metabolite abundances over time in three replicate chemostat cultures inoculated with the NS1 bacterial library. (a) Heatmap of correlation analysis of ASV composition and metabolite levels over time as measured by ^1^H NMR. Correlation coefficients were normalized using a z-score and illustrated by the color gradient. Metabolites and ASVs were ordered based on hierarchical cluster analysis within each dataset. (b, c) Scatter plot of variation over time of (b) *Bacteroides stercoris* or (c) *Bacteroides uniformis* relative abundances and acetate concentrations (mM) profiled by ^1^H NMR. Linear regression analysis and coefficients are shown.

## Data Availability

Data supporting the findings of this study are provided within the supplementary information and in the following public repositories. The 16S rRNA gene sequencing datasets are available in the NCBI database with BioProject ID PRJNA975588 and SRA accession SAMN35530224 to SAMN35530251 and SAMN42898348 to SAMN42898372 (URL access: https://www.ncbi.nlm.nih.gov/bioproject/PRJNA975588/). The metabolomics data from the fecal-derived communities and defined communities were deposited on the MassIVE public repository and are available under the dataset accession numbers MSV000092862 and MSV000092861, respectively [80] (URL access: https://massive.ucsd.edu/). The annotated metadata for the metabolomics data are available in Supplementary Table 18 and Supplementary Table 19. The in-house scripts for performing bioinformatics analyses in this study are available at the GitHub repository https://github.com/DanskaLab/Granato_et_al_Chemostat.
