# Correction: Analysis of early childhood intestinal microbial dynamics in a continuous-flow bioreactor

**DOI:** 10.1186/s40168-026-02424-7

**Published:** 2026-05-08

**Authors:** Alessandra Granato, Simone Renwick, Christopher Yau, Tiffany Kong, Michelle C. Daigneault, Mikael Knip, Emma Allen‑Vercoe, Jayne S. Danska

**Affiliations:** 1 Genetics and Genome Biology, The Hospital for Sick Children, Toronto, ON Canada; 2 Dept. of Molecular and Cellular Biology, University of Guelph, Guelph, ON Canada; 3 Infant Center of Research Excellence, The Larsson-Rosenquist Foundation Mother-Milk, University of California San Diego, La Jolla, San Diego, CA USA; 4 Dept. of Immunology, Faculty of Medicine, University of Toronto, Toronto, ON Canada; 5 Research Program for Clinical and Molecular Metabolism, Faculty of Medicine, University of Helsinki, Helsinki, Finland; 6 Tampere Center for Child Health Research, Tampere University Hospital, Tampere, Finland; 7 Dept. of Medicine Biophysics, Faculty of Medicine, University of Toronto, Toronto, ON Canada

**Correction: Microbiome 12, 255 (2024)**

**https://doi.org/10.1186/s40168-024-01976-w**

Following publication of the original article [1], the author reported that Figures 2 and 3 were missing the connecting lines between the plot.

The incorrect Figures are
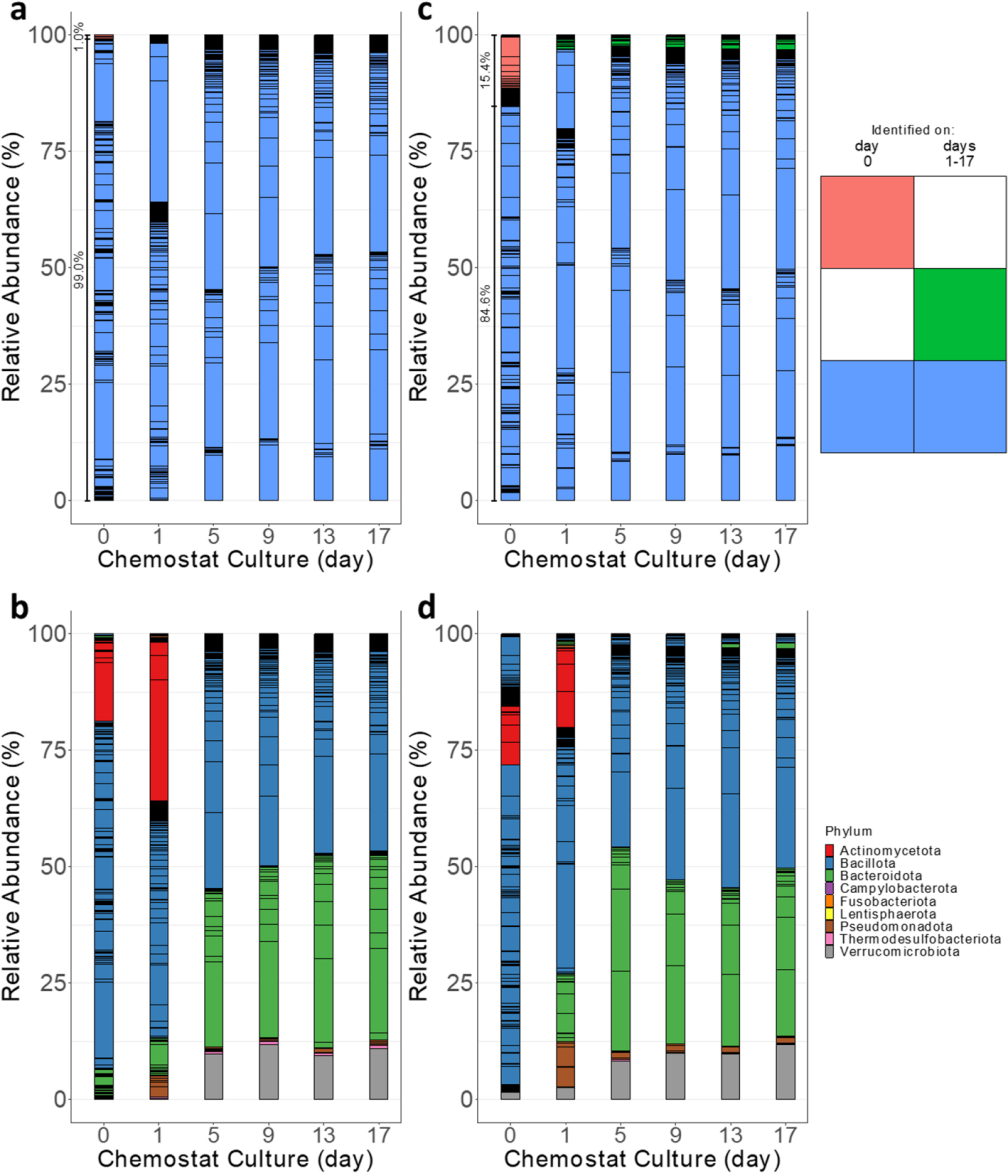



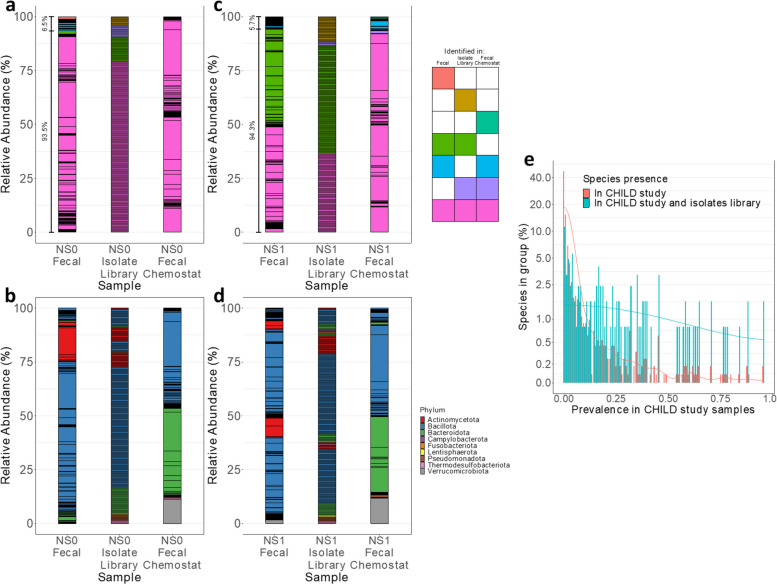


The correct Figures are
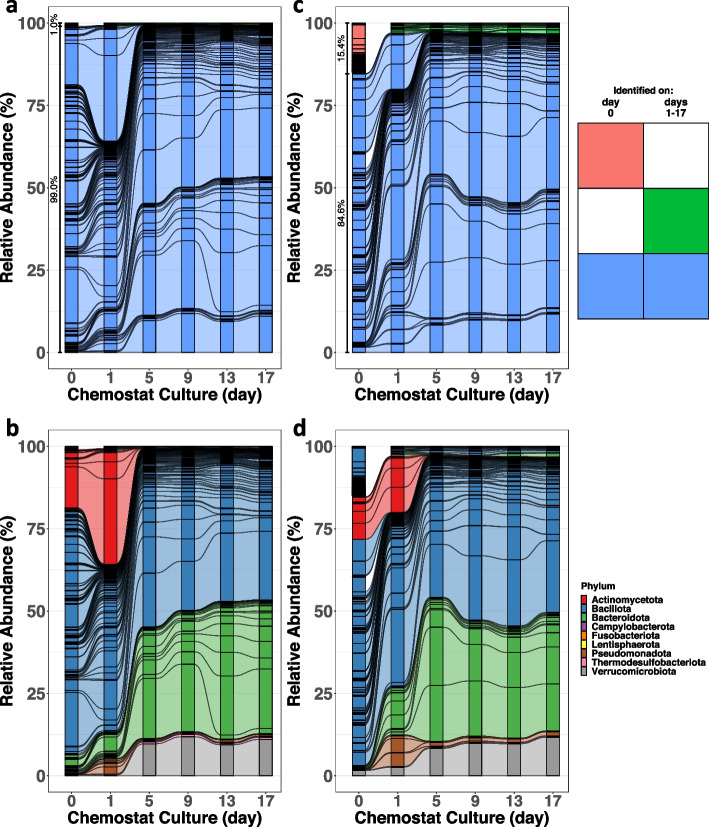



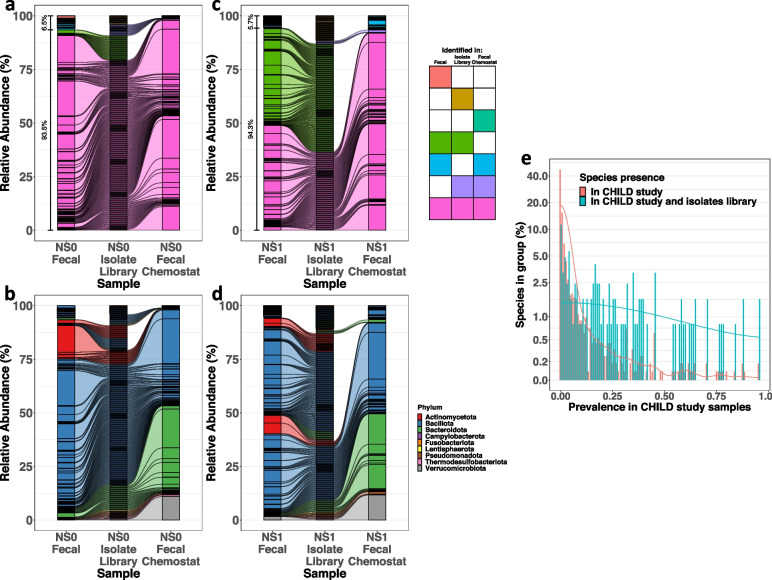


The original article has been updated.
