# Supplementary figures and images for: Analysis of early childhood intestinal microbial dynamics in a continuous-flow bioreactor
Source: Microbiome. 2024 Dec 5;12:255. doi: 10.1186/s40168-024-01976-w (PMC11619690; doi:10.1186/s40168-024-01976-w)

**a**

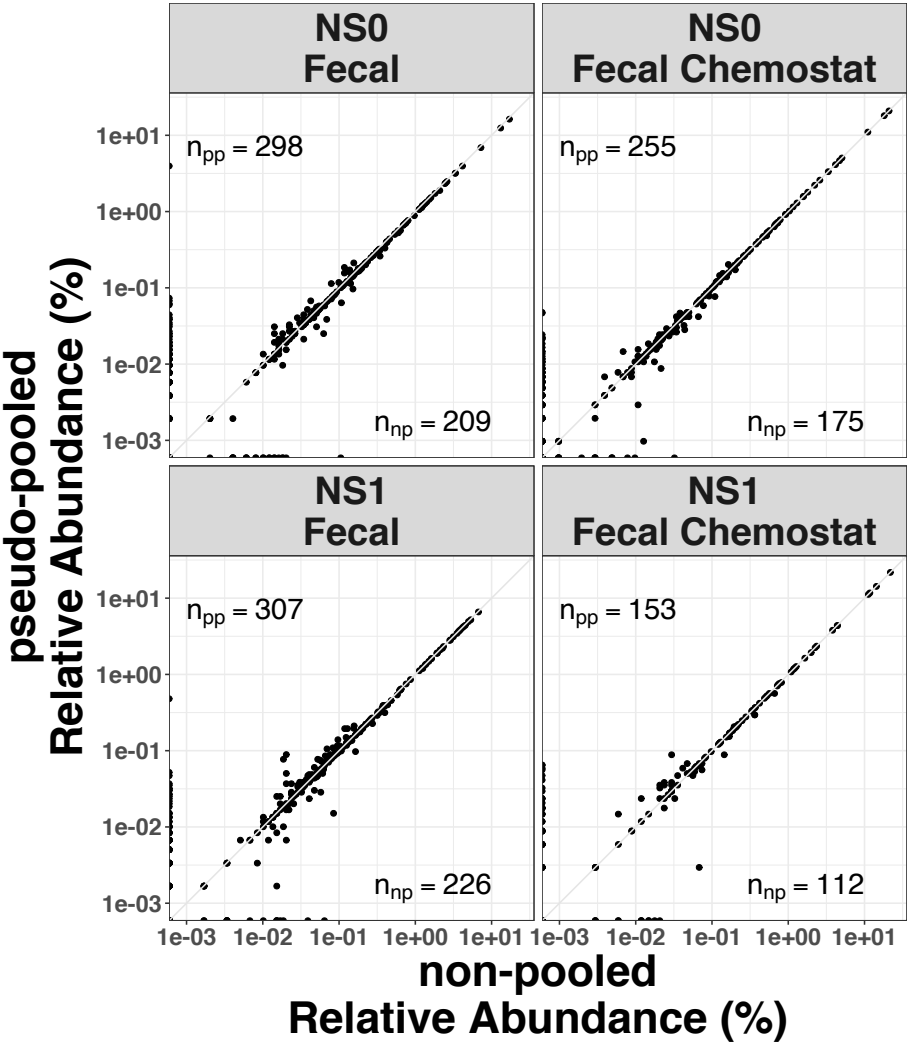

**b**

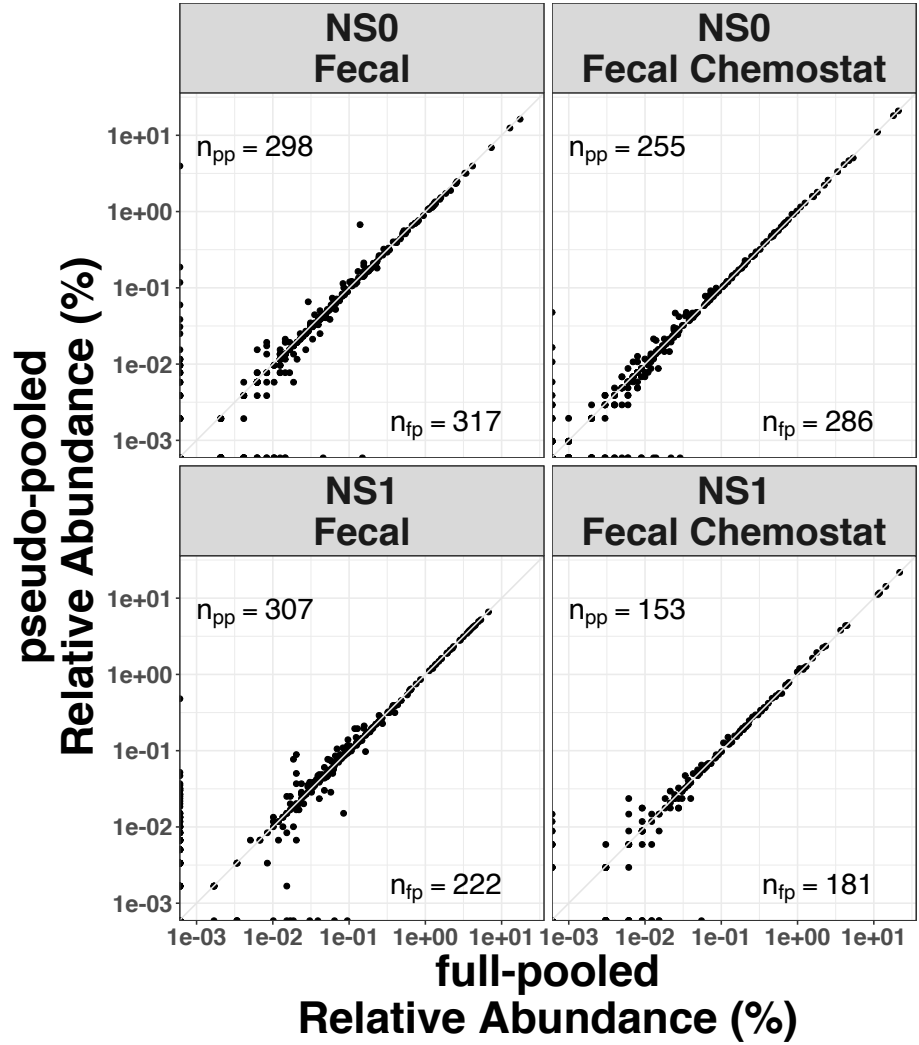

Supplement: Supplementary file 2 — Supplementary Material 2: Figure 1. Relative abundance of ASVs identified in early childhood fecal sample and fecal-derived chemostat culture using DADA2 non-pooled or pseudo-pooled inference methods. DADA2 method aims to accurately reconstruct the exact amplicon sequence variants (ASVs) truly present in a sample from the noisy amplicon sequencing reads [80]. By default (non-pooled method), DADA2 parameters are set to achieve high accuracy by reducing the number of spurious ASV outputs and increasing the specificity. However, the tradeoff for that high specificity is that sensitivity, particularly to rare variants, is reduced by the default non-pooled method. The pseudo-pooled sample inference method allows information to be shared across related samples in a dataset and is particularly effective in longitudinal and inoculation experiments in which samples are taken repeatedly from the same source. This method is expected to improve sensitivity and provide a more accurate description of ASVs at very low frequencies without demanding high computational time. The alternative full pooled sample inference method explicitly infers ASVs across the dataset. This method is also expected to improve sensitivity and provide a more accurate description of ASVs at very low frequencies, at a cost of higher computational time which extends proportionally as a function of the number of samples squared. Here we compare the total number and relative abundance of ASVs identified in two early childhood fecal samples and fecal-derived chemostat cultures using DADA2 non-pooled, pseudo-pooled or full-pooled inference methods. We observe that the pseudo-pooled inference method increased the number of ASVs identified on fecal samples and fecal-derived chemostat cultures. Pseudo-pooling of NS0 samples allowed the identification of additional 81 – 89 ASVs, while in NS1 samples, we observed 41 – 80 new ASVs identified. As expected, most new variants discovered by this method are present in low [file 40168_2024_1976_MOESM2_ESM.pdf]

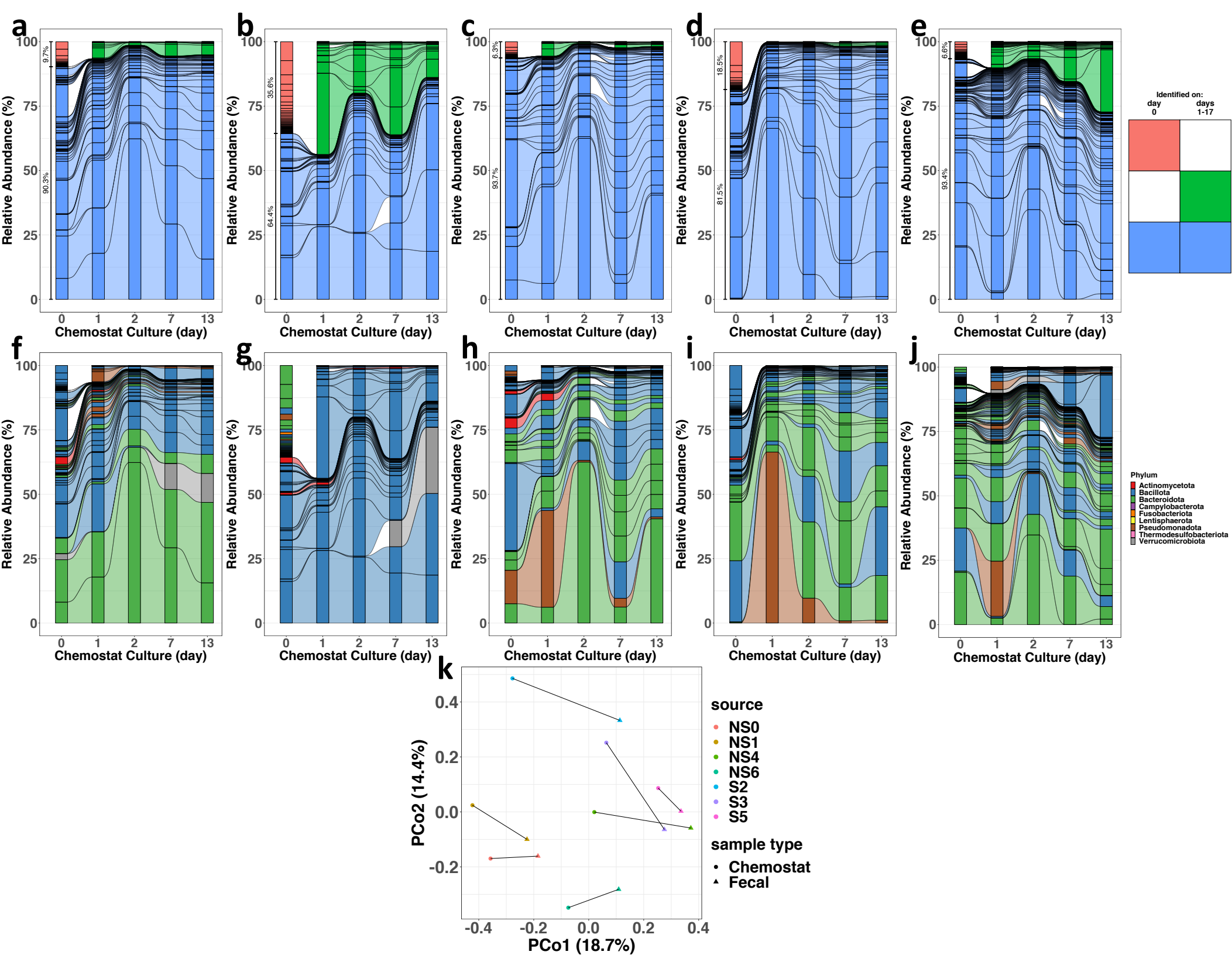

Supplement: Supplementary file 3 — Supplementary Material 3: Figure 2. Five additional fecal samples cultured in the chemostat model generate bacterial communities representative of the donor’s microbiome.Relative abundances of amplicon sequence variants (ASV) that represent bacterial composition were identified by Illumina MiSeq 16S rRNA gene sequencing from fecal samples and longitudinal chemostat communities of an additional five healthy 18-24-month-old participants. (a‑j) Alluvial plots show the relative abundance of ASVs identified on the fecal sample (day 0) and the chemostat culture over time from S2 (a, f), S3 (b, g), NS4 (c, h), S5 (d, i) and NS6 (e, j) individuals. (a-e) ASVs, indicated by colors, are stratified by presence or absence in on the indicated days of culture. The relative abundance of ASVs from the fecal sample found in the chemostat culture is indicated in brackets adjacent to the day 0 bar. (f-j) Colors represent the nine predominant phyla. ASVs are ordered by prevalence categories. (k) Bray-Curtis dissimilarity PCoA of (day 0) fecal sample and day 17 chemostat samples from seven study participants. The first and second principal coordinates are shown. Values in the brackets indicate the % of total variability explained by each principal coordinate. Point shape indicates sample type, and point color indicates the source individual. [file 40168_2024_1976_MOESM3_ESM.pdf]

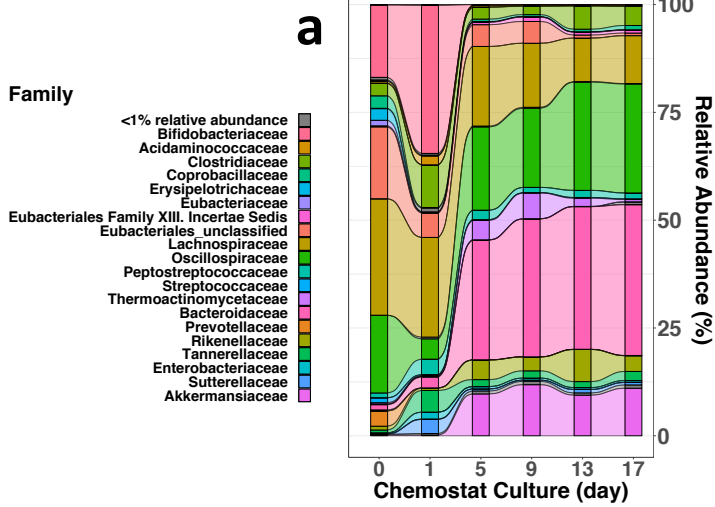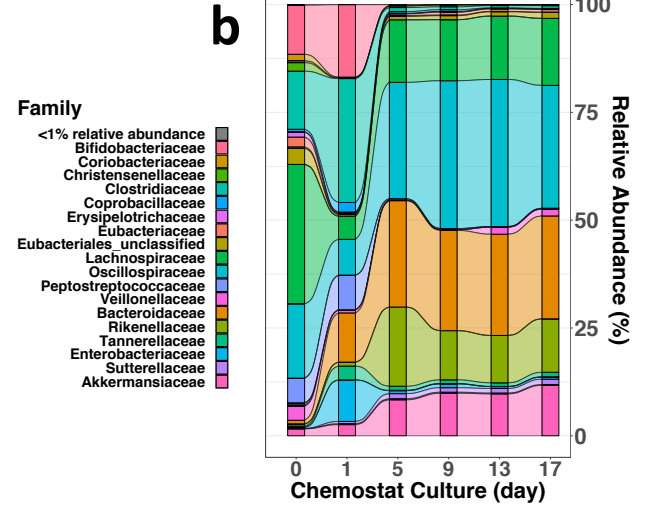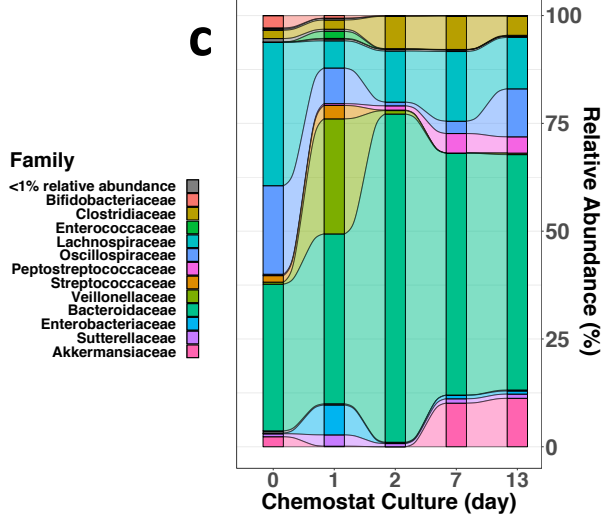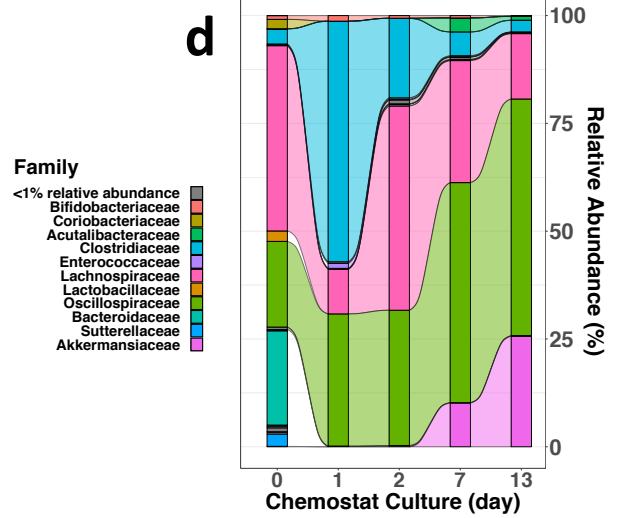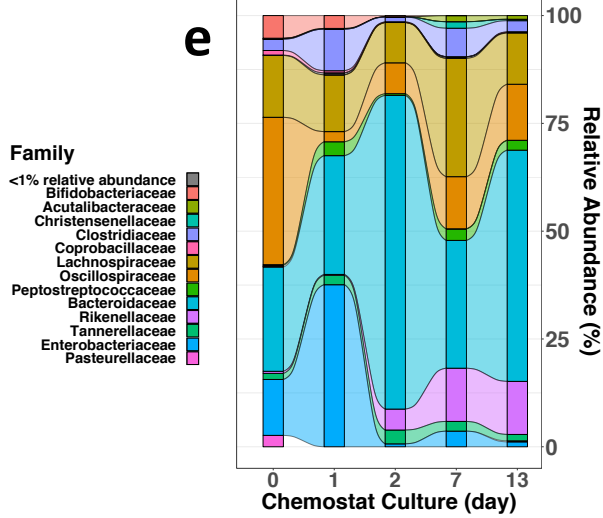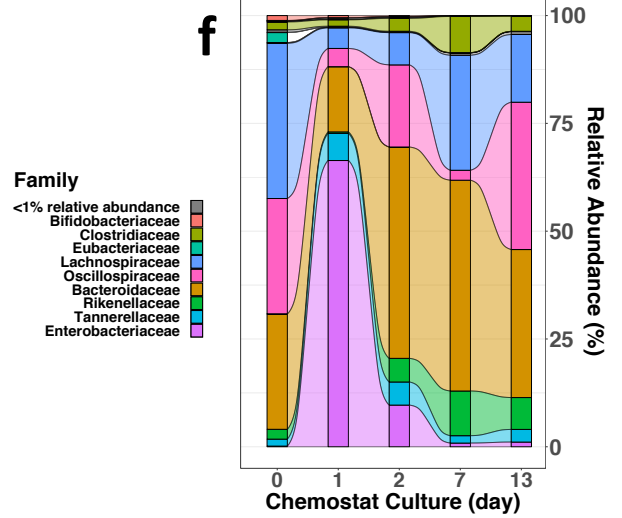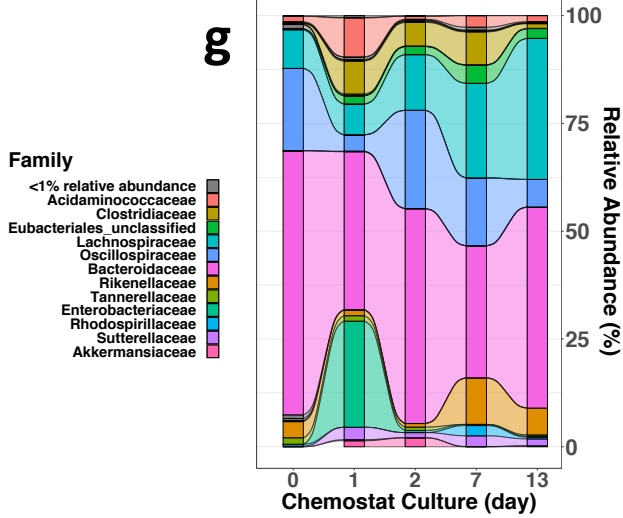

Supplement: Supplementary file 4 — Supplementary Material 4: Figure 3. Family-level collapsed bacterial communities for fecal and fecal-derived chemostats of all seven fecal samples: Relative abundances of amplicon sequence variants (ASV) that represent bacterial composition were identified by Illumina MiSeq 16S rRNA gene sequencing from fecal samples and longitudinal chemostat communities of seven healthy 18-24-month-old participants. ASV relative abundances were collapsed according to highest-likelihood family-level taxanomic classification via BLAST. Alluvial plots show the relative abundance of bacterial families identified on the fecal sample (day 0) and the chemostat culture over time from NS0 (a) and NS1 (b), S2 (c), S3 (d), NS4 (e), S5 (f) and NS6 (g) individuals. Colors represent bacterial families; families are ordered alphabetically by phyla name, then family name. Families appear in each corresponding legend in the same order as in the plot. Families with <1% relative abundance in the samples shown are plotted in grey. [file 40168_2024_1976_MOESM4_ESM.pdf]

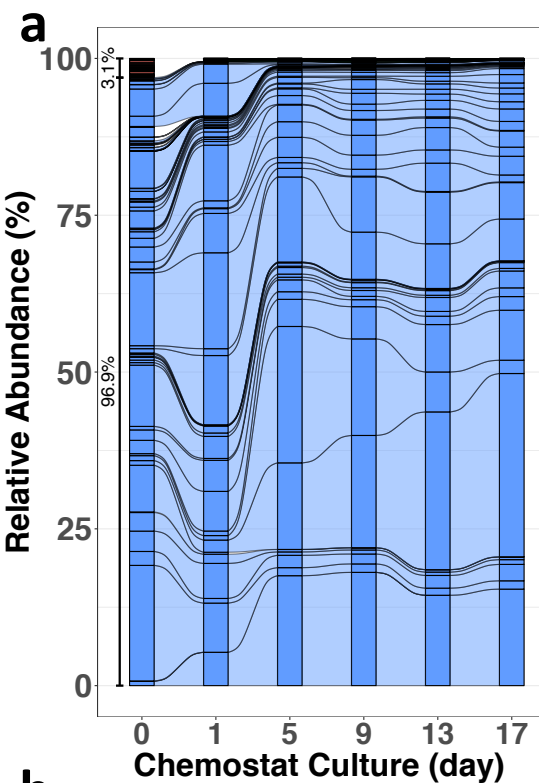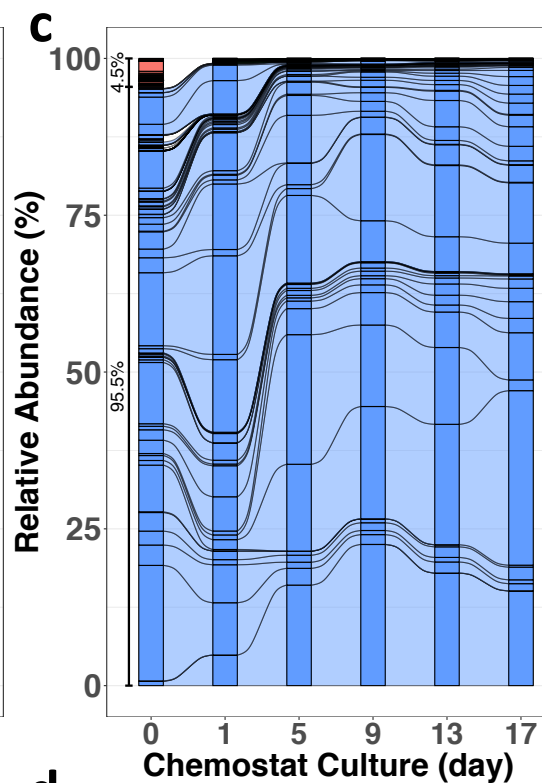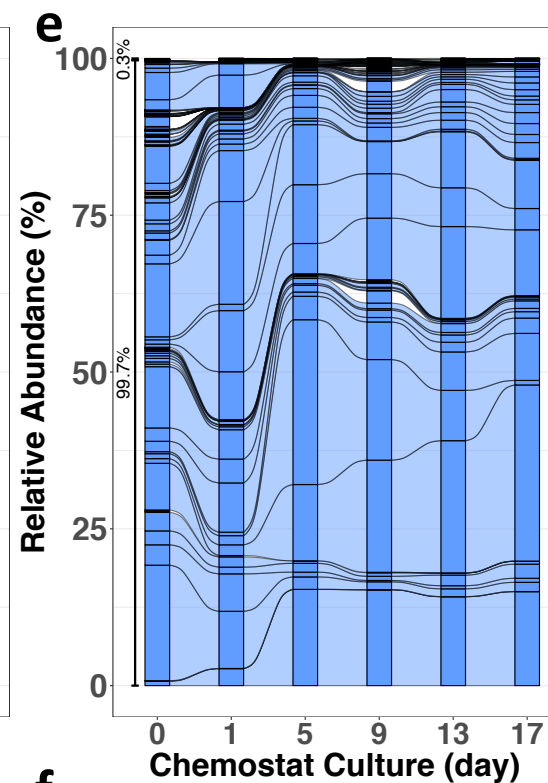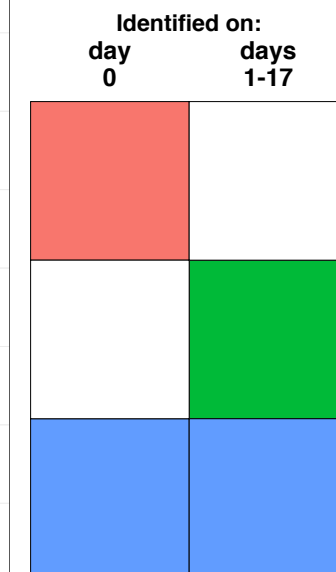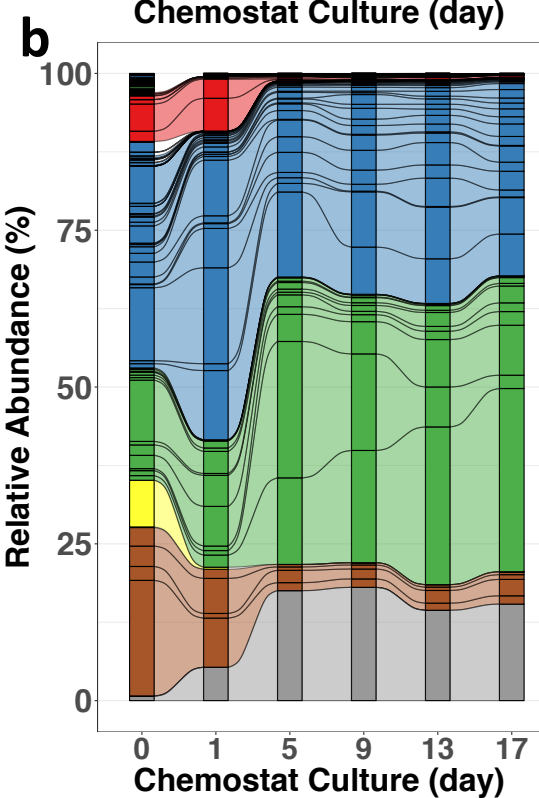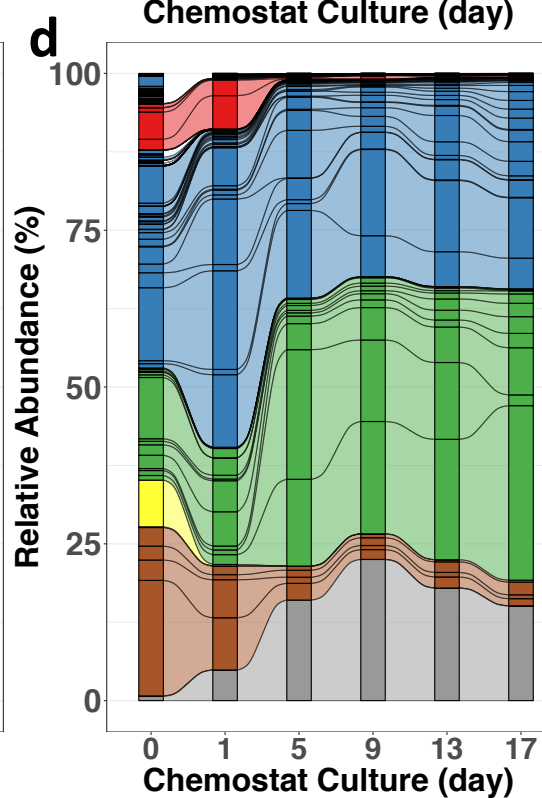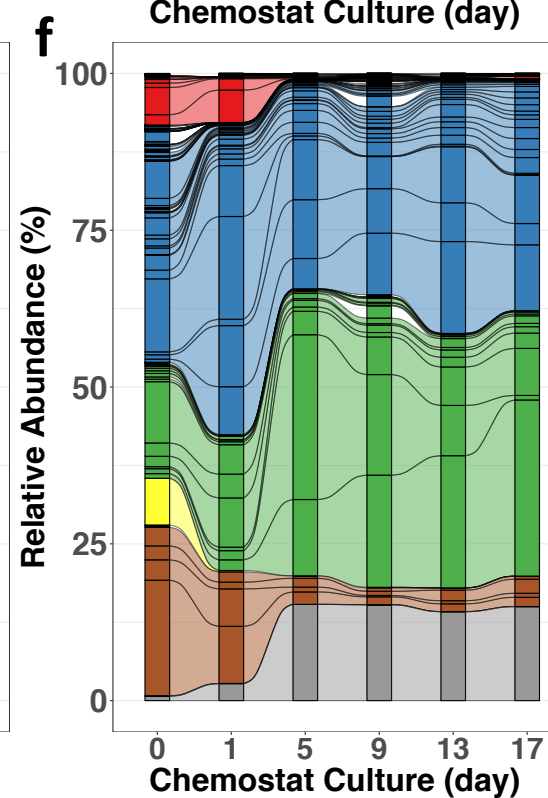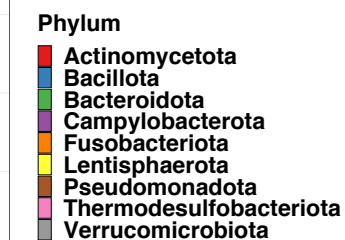

Supplement: Supplementary file 6 — Supplementary Material 6: Figure 5. A library of isolated bacterial strains generates a reproducible bacterial community. (a, c, e) Alluvial plots show the relative abundance of ASVs from the 118 strains defined inoculum (day 0) and following inoculation of three replicate chemostat vessels, sampled over time (key upper right). (b,d,f) ASVs in the inoculum and triplicate cultures stratified by their identification in the culture over time, where colors represent the nine predominant phyla. ASVs are ordered by prevalence categories. [file 40168_2024_1976_MOESM6_ESM.pdf]

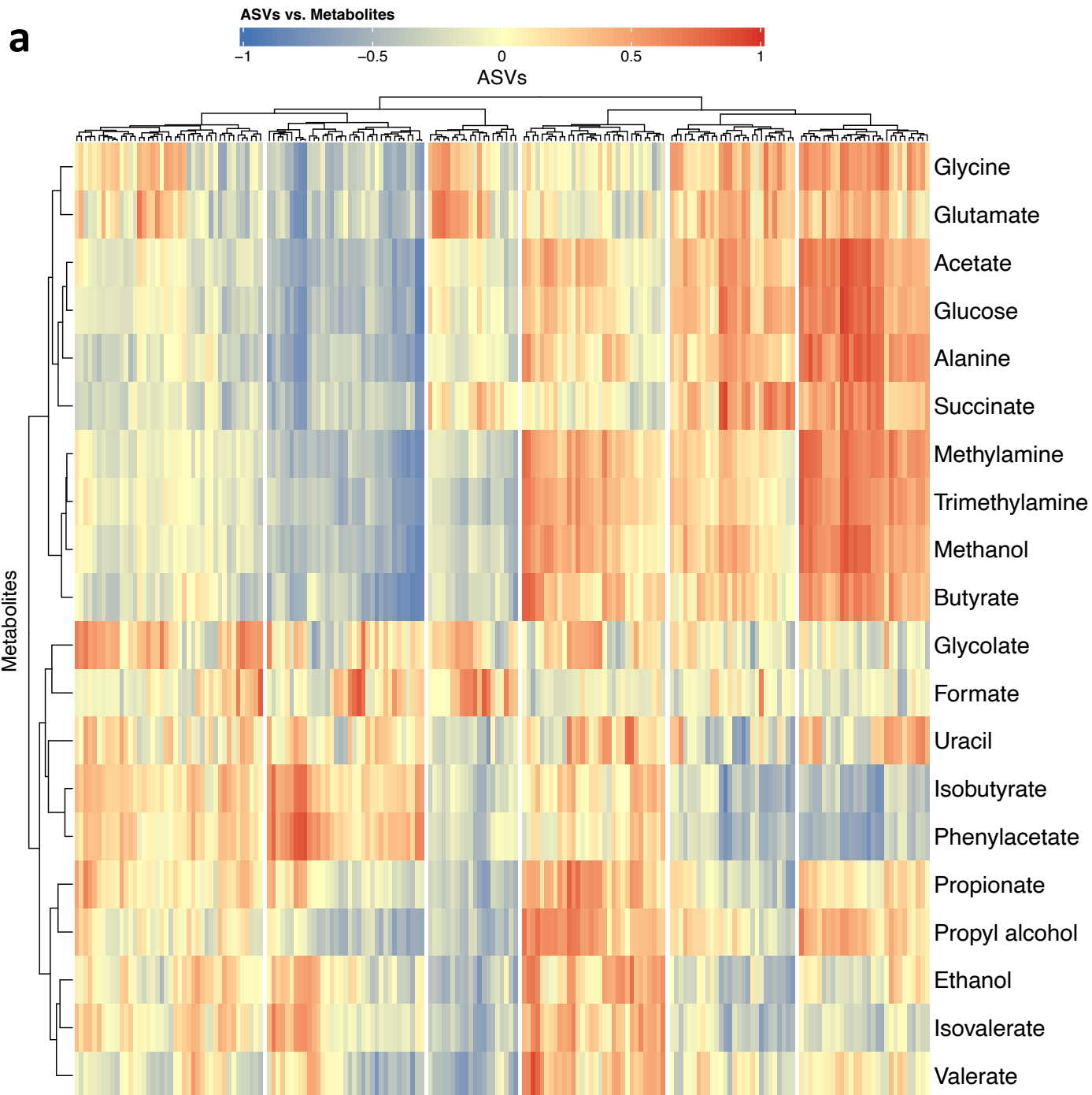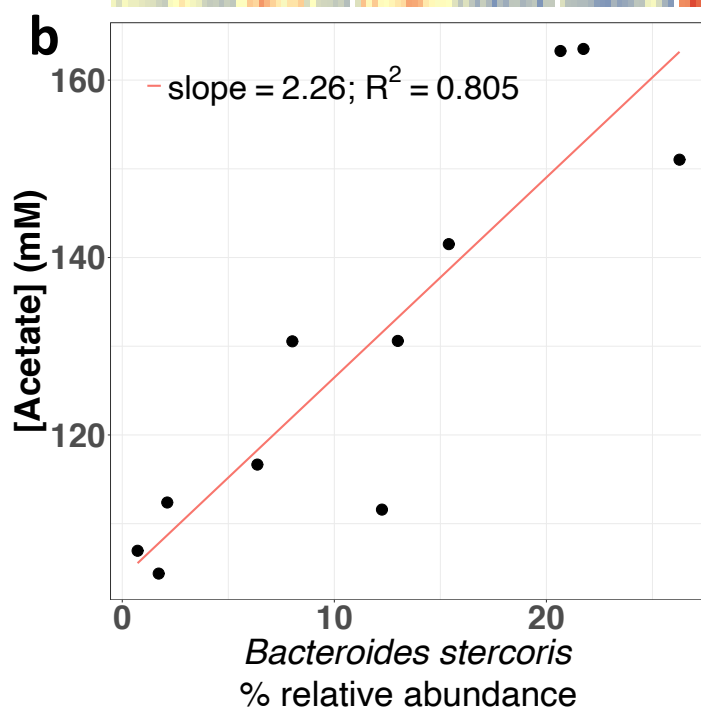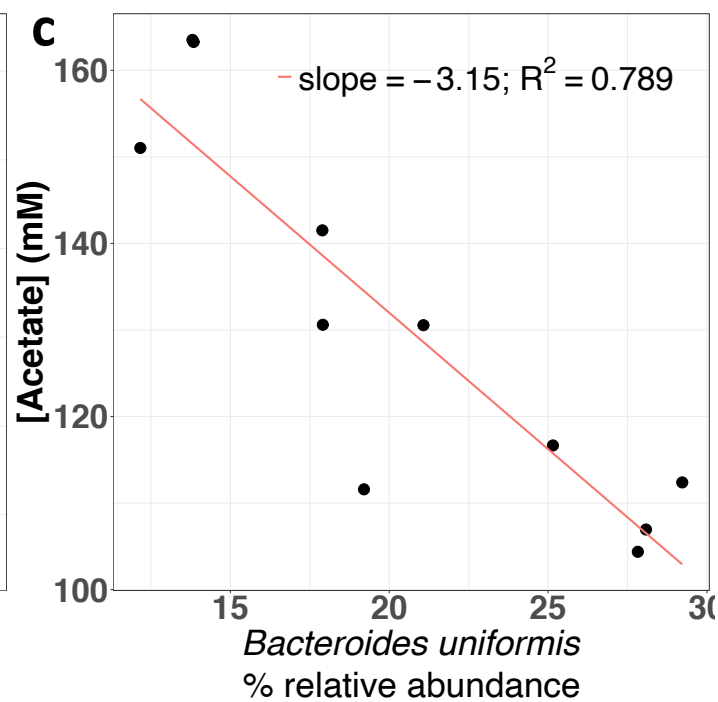

Supplement: Supplementary file 7 — Supplementary Material 7: Figure 6. Association of bacterial taxa and metabolites in replicate chemostat cultures. Correlation analysis of bacterial composition and metabolite abundances over time in three replicate chemostat cultures inoculated with the NS1 bacterial library. (a) Heatmap of correlation analysis of ASV composition and metabolite levels over time as measured by 1H NMR. Correlation coefficients were normalized using a z-score and illustrated by the color gradient. Metabolites and ASVs were ordered based on hierarchical cluster analysis within each dataset. (b, c) Scatter plot of variation over time of (b) Bacteroides stercoris or (c) Bacteroides uniformis relative abundances and acetate concentrations (mM) profiled by 1H NMR. Linear regression analysis and coefficients are shown. [file 40168_2024_1976_MOESM7_ESM.pdf]
