# Supplementary material for: Analysis of early childhood intestinal microbial dynamics in a continuous-flow bioreactor
Source: Microbiome. 2024 Dec 5;12:255. doi: 10.1186/s40168-024-01976-w (PMC11619690; doi:10.1186/s40168-024-01976-w)
Supplement: Supplementary file 5 — Supplementary Material 5: Figure 4. A library of early childhood gut bacterial isolates. Bacterial strains from the fecal-derived chemostat community of NS0 or NS1 individuals were isolated by axenic culture using a variety of media formulations (Suppl. Table 2 ). 80 unique bacterial strains were archived, constituting a library of NS0 sample (Suppl. Table 14 ), and 118 unique bacterial strains were archived, constituting a library of NS1 sample (Suppl. Table 15). (a) Phylogenetic tree of all gut bacterial isolates (species-level) identified by 16S rRNA gene sequencing of NS0 and NS1 libraries. Colored squares indicate phylum. (b) The relative abundance distribution of each isolate species in the fecal samples of CHILD cohort infants at 12 months old is shown (n=842 samples). 16S rRNA gene sequencing dataset from CHILD cohort was obtained at the Sequence Read Archive of NCBI via accession number PRJNA657821. [file 40168_2024_1976_MOESM5_ESM.pdf]

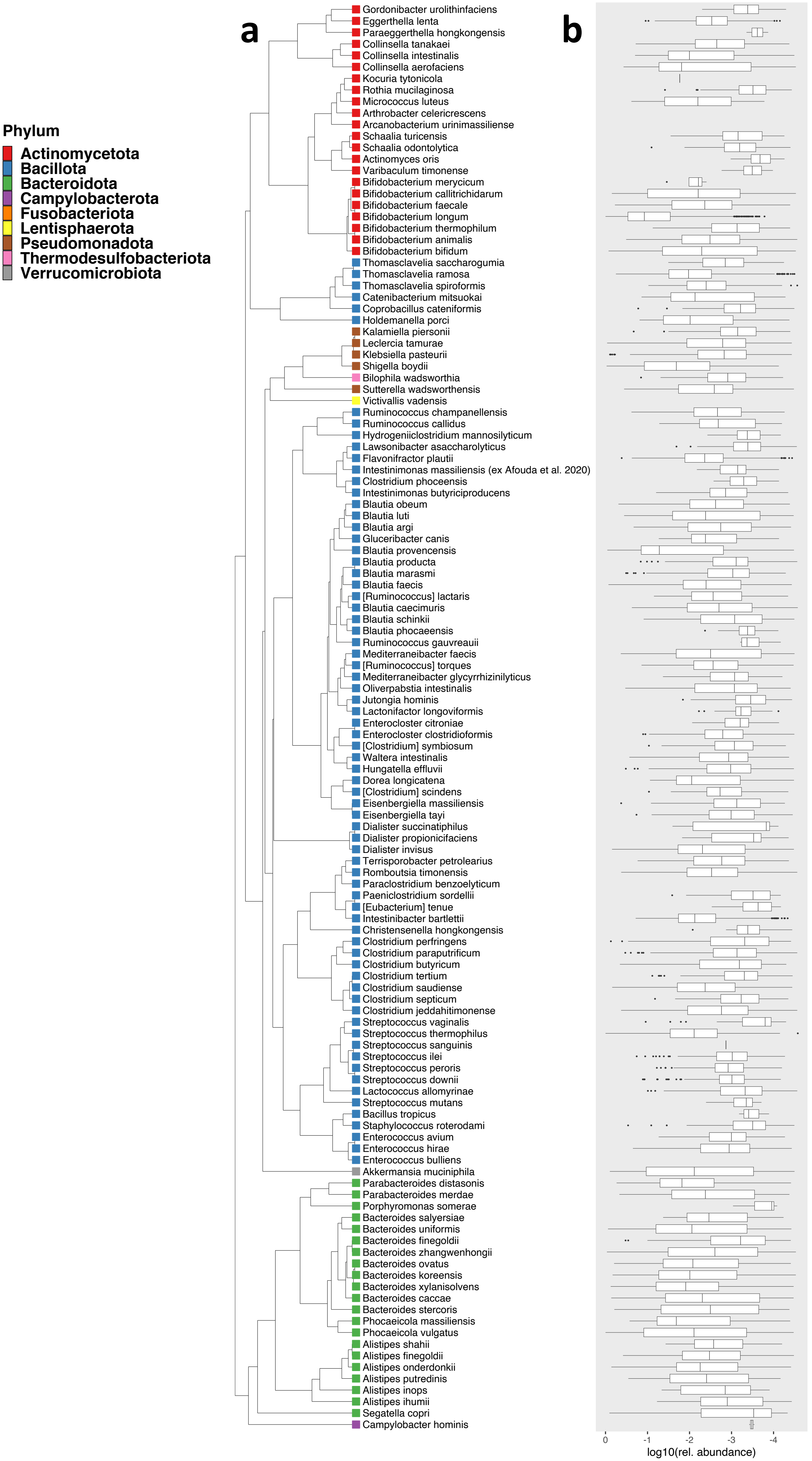

a
b
Phylum
Actinomycetota
Bacillota
Bacteroidota
Campylobacterota
Fusobacteriota
Lentisphaerota
Pseudomonadota
Thermodesulfobacteriota
Verrucomicrobiota
Gordonibacter urolithinfaciens
Eggerthella lenta
Paraeggerthella hongkongensis
Collinsella tanakaei
Collinsella intestinalis
Collinsella aerofaciens
Kocuria tytonicola
Rothia mucilaginosa
Micrococcus luteus
Arthrobacter celericrescens
Arcanobacterium urinimassiliense
Schaalia turicensis
Schaalia odontolytica
Actinomyces oris
Varibaculum timonense
Bifidobacterium merycicum
Bifidobacterium callitrichidarum
Bifidobacterium faecale
Bifidobacterium longum
Bifidobacterium thermophilum
Bifidobacterium animalis
Bifidobacterium bifidum
Thomasclavelia saccharogumia
Thomasclavelia ramosa
Thomasclavelia spiroformis
Catenibacterium mitsuokai
Coprobacillus cateniformis
Holdemanella porci
Kalamiella piersonii
Leclercia tamurae
Klebsiella pasteurii
Shigella boydii
Bilophila wadsworthia
Sutterella wadsworthensis
Victivallis vadensis
Ruminococcus champanellensis
Ruminococcus callidus
Hydrogeniiclostridium mannosilyticum
Lawsonibacter asaccharolyticus
Flavonifractor plautii
Intestinimonas massiliensis (ex Afouda et al. 2020)
Clostridium phoceensis
Intestinimonas butyriciproducens
Blautia obeum
Blautia luti
Blautia argi
Gluceribacter canis
Blautia provencensis
Blautia producta
Blautia marasmi
Blautia faecis
[Ruminococcus] lactaris
Blautia caecimuris
Blautia schinkii
Blautia phocaeensis
Ruminococcus gauvreauii
Mediterraneibacter faecis
[Ruminococcus] torques
Mediterraneibacter glycyrrhizinilyticus
Oliverpabstia intestinalis
Jutongia hominis
Lactonifactor longoviformis
Enterocloster citroniae
Enterocloster clostridioformis
[Clostridium] symbiosum
Waltera intestinalis
Hungatella effluvii
Dorea longicatena
[Clostridium] scindens
Eisenbergiella massiliensis
Eisenbergiella tayi
Dialister succinatiphilus
Dialister propionicifaciens
Dialister invisus
Terrisporobacter petrolearius
Romboutsia timonensis
Paraclostridium benzoelyticum
Paeniclostridium sordellii
[Eubacterium] tenue
Intestinibacter bartlettii
Christensenella hongkongensis
Clostridium perfringens
Clostridium paraputrificum
Clostridium butyricum
Clostridium tertium
Clostridium saudiense
Clostridium septicum
Clostridium jeddahitimonense
Streptococcus vaginalis
Streptococcus thermophilus
Streptococcus sanguinis
Streptococcus ilei
Streptococcus peroris
Streptococcus downii
Lactococcus allomyrinae
Streptococcus mutans
Bacillus tropicus
Staphylococcus roterodami
Enterococcus avium
Enterococcus hirae
Enterococcus bulliens
Akkermansia muciniphila
Parabacteroides distasonis
Parabacteroides merdae
Porphyromonas somerae
Bacteroides salyersiae
Bacteroides uniformis
Bacteroides finegoldii
Bacteroides zhangwenhongii
Bacteroides ovatus
Bacteroides koreensis
Bacteroides xylanisolvens
Bacteroides caccae
Bacteroides stercoris
Phocaeicola massiliensis
Phocaeicola vulgatus
Alistipes shahii
Alistipes finegoldii
Alistipes onderdonkii
Alistipes putredinis
Alistipes inops
Alistipes ihumii
Segatella copri
Campylobacter hominis
0
-1
-2
-3
-4
log10(rel. abundance)
